# Quantitative microbiome profiling reveals the developmental trajectory of the chicken gut microbiota and its connection to host metabolism

**DOI:** 10.1002/imt2.105

**Published:** 2023-04-25

**Authors:** Yuqing Feng, Meihong Zhang, Yan Liu, Xinyue Yang, Fuxiao Wei, Xiaolu Jin, Dan Liu, Yuming Guo, Yongfei Hu

**Affiliations:** ^1^ State Key Laboratory of Animal Nutrition, College of Animal Science and Technology China Agricultural University Beijing China

**Keywords:** chicken gut microbiota, developmental trajectory, quantitative microbiome profiling, relative microbiome profiling, serum metabolites

## Abstract

Revealing the assembly and succession of the chicken gut microbiota is critical for a better understanding of its role in chicken physiology and metabolism. However, few studies have examined dynamic changes of absolute chicken gut microbes using the quantitative microbiome profiling (QMP) method. Here, we revealed the developmental trajectory of the broiler chicken gut bacteriome and mycobiome by combining high‐throughput sequencing with a microbial load quantification assay. We showed that chicken gut microbiota abundance and diversity reached a plateau at 7 days posthatch (DPH), forming segment‐specific community types after 1 DPH. The bacteriome was more impacted by deterministic processes, and the mycobiome was more affected by stochastic processes. We also observed stage‐specific microbes in different gut segments, and three microbial occurrence patterns including “colonization,” “disappearance,” and “core” were defined. The microbial co‐occurrence networks were very different among gut segments, with more positive associations than negative associations. Furthermore, we provided links between the absolute changes in chicken gut microbiota and their serum metabolite variations. Time‐course untargeted metabolomics revealed six metabolite clusters with different changing patterns of abundance. The foregut microbiota had more connections with chicken serum metabolites, and the gut microbes were closely related to chicken lipid and amino acid metabolism. The present study provided a full landscape of chicken gut microbiota development in a quantitative manner, and the associations between gut microbes and chicken serum metabolites further highlight the impact of gut microbiota in chicken growth and development.

## Highlights

- The developmental trajectory of the broiler chicken gut microbiota is revealed by a quantitative microbiome profiling approach.
- The chicken gut microbiota displays gut segment‐specific community types and growth stage‐specific microbes.
- The chicken gut microbes have different colonization abilities, forming different developmental patterns.
- The chicken gut microbiota development is associated with the chicken serum metabolite changes.

## Graphical abstract

In this study, we revealed the developmental trajectory of the broiler chicken gut bacteriome and mycobiome by combining high‐throughput sequencing with a microbial load quantification assay. Furthermore, we provided links between the absolute changes in chicken gut microbiota and their serum metabolite variations. The present study provided a full landscape of chicken gut microbiota development in a quantitative manner, and the associations between gut microbes and chicken serum metabolites further highlight the impact of gut microbiota in chicken growth and development.

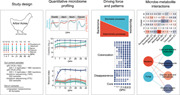

## INTRODUCTION

The demand for poultry products has grown exponentially in recent decades, and chicken meat has become the most consumed animal meat worldwide [1]. The chicken gut microbiota strongly influences immunity, nutrient digestion, and feed efficiency [2], which highlights the importance of the gut microbiota in chicken health and productivity. Similar to other animals, the chicken gut microbiota primarily consists of bacteria, archaea, fungi, and viruses [3]. Many studies used culture‐dependent and culture‐independent methods to investigate chicken gut microbial composition and function [4, 5, 6]. However, most studies mainly focused on the characterization of the chicken gut bacteriome, with less effort to reveal the fungal community that also contributes to chicken digestion and nutrient supply [7]. Additionally, more studies are concerned about the microbiota in the chicken lower gastrointestinal tract (GIT) than in the upper GIT.

The assembly and succession of microbiota within the host GIT have attracted more attention in recent years [8, 9, 10, 11]. The development of gut microbiota during early life has a far‐reaching impact on host health, including stimulating the development of the immune system and the maturation of immune cells, endocrine system homeostasis, and host metabolism and development [12]. Gut bacterial community changes are age‐associated in chickens. Proteobacteria dominate the cecal microbiota in the early stage, followed by Firmicutes in the later stage [13, 14]. *Lactobacillus* species are the most abundant bacteria in the ileum during the entire production period [15, 16, 17, 18]. Development of the chicken intestinal mycobiome was rarely considered until a very recent work revealed that the mycobiome was more diverse in the upper GIT than the lower GIT, and no apparent trend of succession was observed up to 42 days of age [19]. Importantly, this study compared the absolute abundance (AA) of the fungal and bacterial communities and found that fungi accounted for a small proportion of the chicken gut microbiota.

Compared with relative abundances (RAs) that are used to profile the proportions of different microbial taxa within a community across time and space, quantitative microbiome profiling (QMP) concerns changes in AA and determines whether a species thrives or withers over time. Therefore, QMP allows us to elucidate the real community dynamics, which facilitates the identification of biotic and abiotic forces that shape a microbial community and a better correlation between gut microbiota features and host changes [20]. Additionally, the microbial load itself may be an identifier of an ecosystem configuration, such as in health and disease statuses [21]. Human microbiome studies have used QMP to identify microbiome markers associated with pathophysiological manifestations and disease diagnosis in patients with inflammatory bowel disease [22]. Absolute quantification‐based microbe–microbe interactions revealed that the assembly dynamics in preterm infants were likely driven by context‐dependent interactions between specific microbes [20]. Different approaches have been developed to quantify microbial loads, such as microbial DNA content, quantitative polymerase chain reaction (PCR), flow cytometry, and microbial spike‐in techniques [23]. Compared with relative microbiome profiling (RMP), QMP transforms sequencing data into an absolute microbiome abundance matrix with an adjustment of the absolute number of taxa before the downstream analyses.

Previous studies have investigated the development of chicken gut microbiota using relative qualification of 16S amplicon sequences [18]. However, the absolute amount‐based development trajectories of bacteria and fungi across the upper and lower chicken GIT are not fully understood. The present study investigated the succession of chicken gut microbial communities from the duodenum, jejunum, ileum, and cecum during a 42‐day production period using the QMP approach. We also used untargeted metabolomes to characterize the metabolite changes in chicken serum over time and constructed potential connections between the development of gut microbiota and chicken metabolism.

## RESULTS

### Abundance and diversity of the chicken gut microbiota reach a plateau at 7 days posthatch (7 DPH)

To investigate the developmental trajectory of the microbial community in the broiler chicken gut throughout the production period (1–42 days of age), we examined 320 luminal content samples from a total of 80 birds and investigated the succession of bacteria and fungi in four gut segments using the QMP method. Because the primers based on the V3–V4 regions of the 16S rRNA gene detect bacteria and archaea, we also profiled the dynamics of archaea. We found that there was a low prevalence (less than 15.0%) of archaea in the samples, and no archaea were found in the ileum (Supporting Information: Figure S1A). Moreover, in samples with the presence of archaea, the numbers of bacteria and fungi were ~4 and ~1–3, respectively, which were orders of magnitude larger than archaea (Supporting Information: Figure S1B). These results suggest that archaea are scarce in the chicken gut, which is consistent with our previous findings [24]. Therefore, we focused on analyzing the bacteriome and mycobiome.

**Figure 1:**
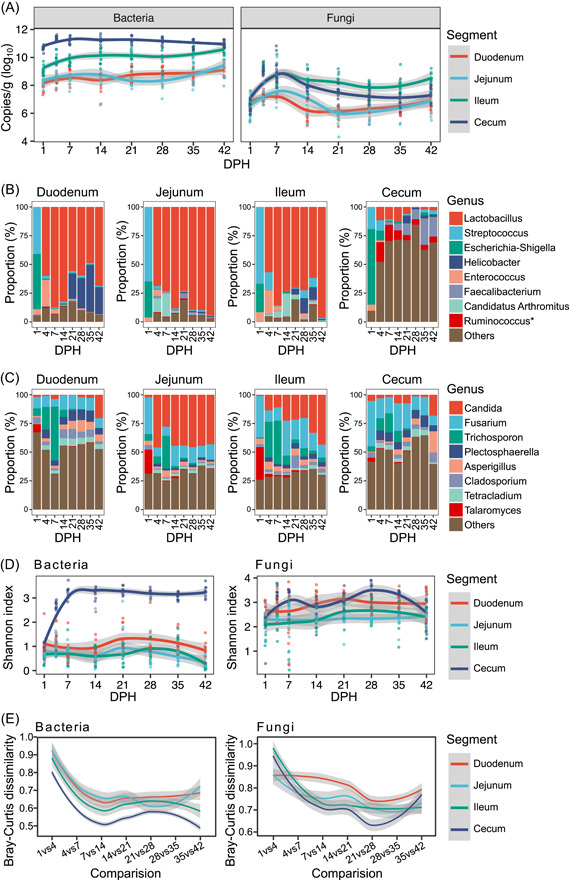
Abundance and diversity changes of the quantitative chicken gut microbiota. (A) Absolute abundance dynamics of bacteria and fungi in the four segments of broiler chickens. Bacterial (B) and fungal (C) community composition of the broiler gut microbiota at the genus level. *, torques group. (D) Shannon index of the samples during the 42 DPH. (E) Pairwise comparison of Bray–Curtis dissimilarity of samples from adjacent time points. DPH, days posthatch.

We found that the AA of bacteria and fungi reached a plateau at 7 DPH, and the bacterial load seemed to increase along the broiler gut. The fungal load was higher in the ileum than in the cecum, especially after 7 DPH (Figure 1A). The AA of bacteria increased over time in the first three gut segments, but fungi exhibited an increasing then decreasing trend in all four gut segments. Composition analysis indicated that there were a total of 25 phyla of bacteria and 9 phyla of fungi in the gut microbiota. At the phylum level, Firmicutes and Ascomycota were the predominant phyla of bacteria and fungi, respectively (Supporting Information: Figure S2). The bacterial phylum Bacteroidetes began to expand in the cecum at 14 DPH, and the fungal phylum Basidiomycota showed a decreasing trend with age in most gut segments. Notably, the newly hatched broilers harbored a large proportion of Proteobacteria in all four segments. At the genus level, *Lactobacillus* was the predominant bacterial genus in the first three gut segments, but not in the cecum, at 4 DPH and thereafter (Figure 1B). The genera *Streptococcus* and *Escherichia*‐*Shigella* occupied the entire digestive tract at 1 DPH. The fungal communities were more complex in composition. *Candida*, *Fusarium*, and *Trichosporon* generally dominated all gut segments during the entire 42 days of age, and *Candida* seemed more adapted to the jejunum and ileum (Figure 1C). The alpha diversity of bacterial communities was much higher in the cecum than in the other three gut segments, which was likely due to the dominance of *Lactobacillus* in the foregut. The diversity of fungal communities was relatively less variable over time and space (Figure 1D). However, the diversity of bacterial and fungal communities in the cecum increased dramatically during the first 7 days, which indicated rapid colonization of gut microbes at this time. The microbiota assembly process and changes in microbial diversity were further reflected by the differences in the community structures in different gut segments (Supporting Information: Tables S4 and S5 and Figure S3, adjusted *p* < 0.05). The pairwise Bray–Curtis dissimilarity between samples from adjacent time points further indicated that more changes in bacterial and fungal communities occurred in the first 7 days (Figure 1E). Taken together, these results suggested different microbiota assembly processes in different segments of the broiler chicken gut, and the microbial abundance and diversity reached a relatively stable state at 7 DPH during the development of the gut microbiota.

### RMP biases chicken gut microbiota development and microbial interactions

**Figure 2:**
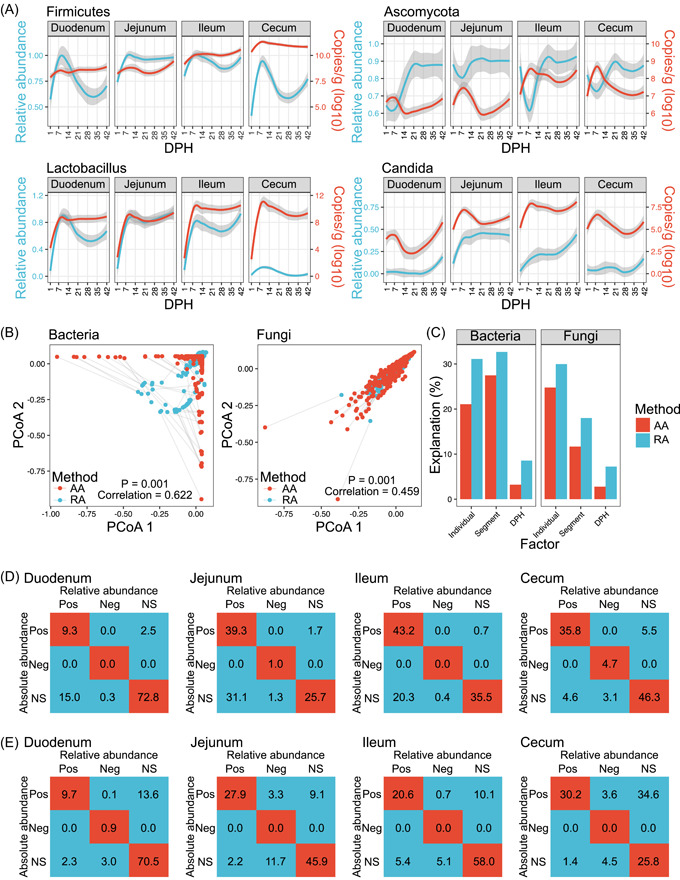
Differences in QMP and RMP in profiling the chicken gut microbiota. (A) Changes in the absolute abundance and relative abundance of the main phyla (Firmicutes and Ascomycota) and the main genera (*Lactobacillus* and *Candida*) in the broiler gut microbiota. Red lines: absolute abundance of taxa; blue liens: relative abundance of taxa. (B) Procrustes analyses of the correlation between absolute abundance and relative abundance for bacteria and fungi. Red nodes: absolute abundance of bacterial or fungal communities; blue nodes: relative abundance of bacterial or fungal communities. (C) The percentage of the variance of the gut microbiota is explained by the factors of individuals, segments, and time. (D) Consistency of the correlations between the taxa based on absolute abundance and relative abundance in bacteria. (E) Consistency of the correlations between the taxa based on absolute abundance and relative abundance in fungi. Spearman's correlations with adjusted *p* values less than 0.05 were considered significant. The numbers in the cells are the ratio of each pair of correlations in different categories. AA, absolute abundance; DPH, days posthatch; Neg, negative correlation; NS, nonsignificant; PCoA, principal coordinate analysis; Pos, positive correlation; QMP, quantitative microbiome profiling; RA, relative abundance; RMP, relative microbiome profiling.

Because the microbial loads changed over time and space in host guts, RMP hindered attempts to construct associations between gut microbes and host features [21]. To identify differences between the QMP and RMP approaches in profiling chicken gut microbiota, we first examined the developmental trajectories of the predominant taxa at the phylum and genus levels. Significant differences in microbial development were observed at both levels. For example, the AA of the bacterial phylum Firmicutes was relatively stable in the four gut segments. However, the RA of this phylum exhibited greater variations, especially in the duodenum and cecum (Figure 2A). The fungal phylum Ascomycota showed the opposite development trend in QMP and RMP quantitation across the four gut segments. Although a similar trend of changes was found at the genus level for the abundance of *Lactobacillus* in the two methods, QMP indicated that the absolute number of this genus was similar in different gut segments after 7 DHP, but RMP showed that its abundance was extremely low in the cecum. Similar results were found for the representative fungal genus *Candida*. Procrustes analyses indicated that AA and RA were correlated with each other for bacterial and fungal communities (*p* < 0.001). However, the correlation coefficients were only 0.622 and 0.459 for the bacteriome and mycobiome, respectively (Figure 2B). Compared with AA, RA exaggerated the proportion of variation explained by factors of individual, segment, and DPH (Figure 2C).

Co‐occurrence networks based on microbial abundance correlation are widely used in gut microbiome analysis to deduce and interpret microbial interactions [21]. To examine the impacts of QMP and RMP on revealing microbial interactions, we analyzed Spearman's correlation at the genus level in different gut segments. A large number of significantly covarying genus pairs were found in both the QMP and RMP networks (Supporting Information: Figure S4). The consistencies of QMP and RMP microbial abundance correlations (Pos + Neg + NS correlation) for the bacteriome in the four gut segments (duodenum, jejunum, ileum, and cecum) were 82.1%, 66.0%, 78.7%, and 86.8%, respectively, and 81.1%, 73.8%, 78.6%, and 56.0%, respectively, for the mycobiome (Figure 2D,E). The RMP approach was prone to overestimate the positive correlations found using the QMP approach for the bacteriome (402 vs. 196 in the duodenum, 1164 vs. 678 in the jejunum, 1050 vs. 725 in the ileum, and 668 vs. 683 in the cecum) and missed positive correlations for the mycobiome (400 vs. 775 in the duodenum, 997 vs. 1338 in the jejunum, 865 vs. 1044 in the ileum, and 1048 vs. 2270 in the cecum).

Overall, RMP quantitation ignored the absolute growth or decline of microbes and failed to detect real microbial changes in the chicken gut. Therefore, this quantitation biased the developmental trajectory of the gut microbiota and the microbial interactions within it.

### Ecological deterministic and stochastic processes shape the segment‐specific community types

**Figure 3:**
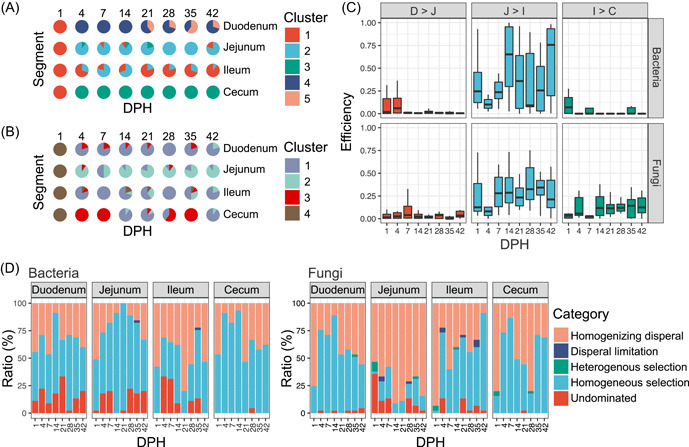
Clusters of bacterial and fungal communities associated with the segment and the selection pressure. DMM clustering of bacterial (A) and fungal (B) communities. Different colors represent different DMM clusters. (C) Relative contributions of upper microbial communities to the adjacent lower microbial communities. (D) Relative importance of different ecological processes (heterogeneous selection: βNTI < −2, homogeneous selection: βNTI > 2, dispersal limitation: |βNTI| < 2 and RC_Bray_ > 0.95, homogenizing dispersal: |βNTI| < 2 and RC_Bray_ < −0.95, and undominated: |βNTI| < 2 and |RC_Bray_| < 0.95) along the broiler gut microbiota. βNTI, β‐nearest taxon index; DMM, Dirichlet multinomial mixture; DPH, days posthatch; RC_Bray_, Bray–Curtis‐based Raup–Crick Index.

To further investigate the dynamic changes in broiler chicken gut microbiota using the QMP quantitation method, we first used Dirichlet multinomial mixture (DMM) to cluster community types in different gut segments. We identified five and four community types for bacterial and fungal communities, respectively. The genera *Lactobacillus*, *Streptococcus*, and *Faecalibacterium* dominated the bacterial community types, and the genera *Tetracladium*, *Plectosphaerella*, *Fusarium*, and *Candida* dominated the fungal community types (Supporting Information: Figure S5A,B). These community types exhibited significant differences in microbial loads, Shannon indexes, and microbial compositions (*p* < 0.05, Supporting Information: Figure S5C–H) and had different spatial and temporal distributions (Figure 3A,B). At 1 DPH, only bacterial community type 1 and fungal community type 4 were present in all gut segments. From 4 DPH onward, bacterial community types 4 and 5 were common in the duodenum, types 1 and 2 were prevalent in the jejunum and ileum, and type 3 predominated in the cecum. For the fungal community, type 2 was more common in the jejunum, and type 3 showed a high frequency in the cecum. These observations suggested that the chicken gut microbiota quickly formed segment‐specific community types after hatching.

The segment‐specific microbial distribution prompted us to investigate the effects of the microbial community in the anterior gut segment on the microbial population in posterior gut segments. We used fast expectation‐maximization microbial source tracking (FEAST) to investigate the source of the microbiota in the latter three gut segments (jejunum, ileum, and cecum). We found limited microbial transmission from the duodenum to the jejunum and from the ileum to the cecum. Only the jejunal microbiota contributed approximately half of the ileal microbiota (Figure 3C). We used an ecological model applied in a previous study [25] to examine the internal driving forces of the segment‐specific community types. The results indicated that deterministic processes (56.7%) played a more important role than stochastic processes (43.3%) in bacterial communities, and stochastic processes (55.1%) played a more important role than deterministic processes (44.9%) in fungal communities (Figure 3D). Homogeneous dispersal (32.7% for bacteria and 50.3% for fungi) and homogeneous selection (56.7% for bacteria and 44.0% for fungi) were the two processes that governed the assembly of bacterial and fungal communities. Higher proportions of homogeneous selection in bacterial communities may have led to similar and stable community structures in each segment. The fungal communities were in a state of unpredictable and disordered community composition under homogeneous dispersal, except for fungal communities in the jejunum. High dynamic changes in the fungal community in the chicken gut were also observed in a more recent study [19].

### Chicken gut microbes have different colonization abilities

**Figure 4:**
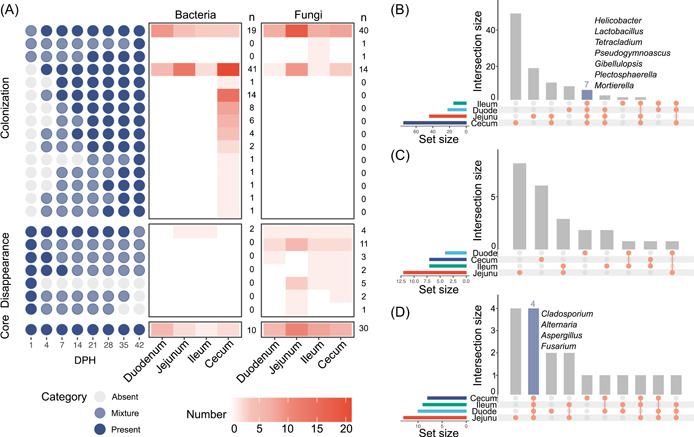
Longitudinal occurrence patterns and variants of microbial genera across the four gut segments. (A) Summary of the occurrence patterns matched the three categories (core, disappearance, and colonization) in the four segments. Dark blue points represent the presence of taxa (*n* ≥ 9); gray points represent the absence of taxa; and light blue points represent the transition between “presence” and “absence” throughout the 42 days. The color of the cell represents the counts of each pattern. The UpSetR plot highlights the intersection of the genera colonized (B), disappeared (C), and presented (D) in the four segments. DPH: days posthatch.

We examined the order and timing of bacterial and fungal genera's arrival during the 42‐day period. The appearance orders and timing of the broiler gut microbiota members in different segments are shown in Supporting Information: Figure S6 (bacteria) and Figure S7 (fungi). The number of longitudinal occurrence patterns in the four segments varied from 71 to 168 (Supporting Information: Table S6). Most of the patterns co‐existed among the sampled chicken population (mixture) throughout the 42‐day period, which suggested that a large proportion of microbes were not stable in different individuals. However, stable patterns were found in specific gut segments. For example, two patterns of bacteria containing the most genera (21 and 14) commonly existed in the cecum (Supporting Information: Figure S6). Genera in these two patterns showed rapid colonization (at 4 and 7 DPH) and became stable residents of the population. We further assigned all of the occurrence patterns that exhibited regular changes (*n* = 23) into three categories: “colonization,” “disappearance,” and “core” (Figure 4A and Supporting Information: Table S7). There were 15 patterns (107 genera in total) in the “colonization” category, which were not observed after hatching but appeared at later time points. Notably, two bacterial genera (*Helicobacter* and *Lactobacillus*) and five fungal genera (*Tetracladium*, *Pseudogymnoascus*, *Gibellulopsis*, *Plectosphaerella*, and *Mortierella*) in this category were shared among the four gut segments (colonization at 1 DPH and stable thereafter) (Figure 4B and Supporting Information: Table S8). Seven patterns containing 24 genera were assigned to the “disappearance” category, which appeared at 1 DPH and disappeared at later time points. Notably, more fungal genera (*n* = 22) exhibited this pattern than bacterial genera (*n* = 2), which suggests that some bacteria have more competitive advantages in the colonization process. No shared genus in this category was found among the four gut segments (Figure 4C and Supporting Information: Table S9). One pattern containing 18 genera belonged to the “core” category that was present during the entire 42‐day period. The AA of these 18 genera was relatively stable throughout the time span in specific gut segments or the whole GIT (Supporting Information: Figure S8), which suggests a high robustness and self‐adjusting capacity. Among the 18 genera, four fungal genera, *Cladosporium*, *Alternaria*, *Aspergillus*, and *Fusarium*, existed in all four gut segments (Figure 4D and Supporting Information: Table S10), which suggests that these fungi possess greater niche‐adaptation ability than the bacteria and other fungi.

### Co‐occurrence networks reveal more positive associations among microbes in the chicken gut

**Figure 5:**
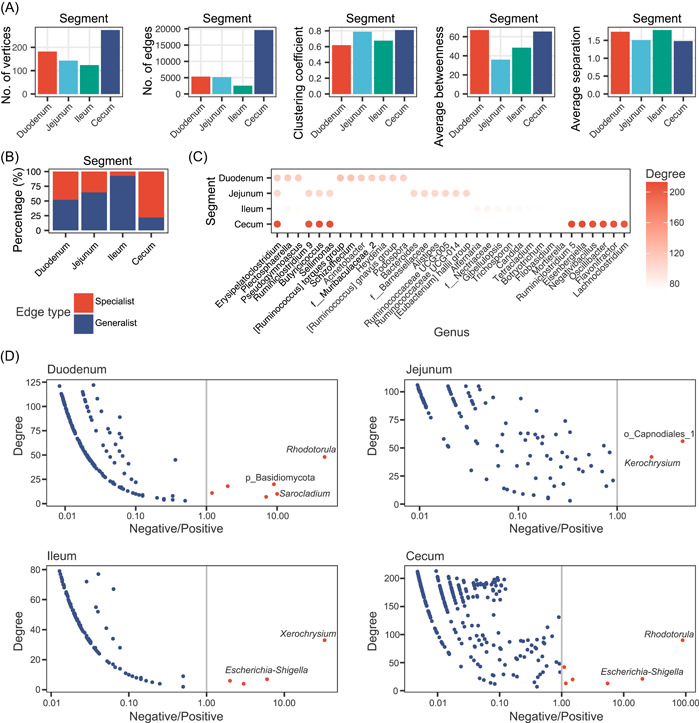
Microbial co‐occurrence networks in the four gut segments. (A) Network topology of microbial networks inferred from absolute microbiome profiling. (B) Proportions of generalist edges and specialist edges in the four microbial networks. (C) Top 10 key taxa in the four gut segments. The color of each point represents the mean degree of each taxon. The blank position represents that this taxon is not a key taxon as determined by the degree in each network. (D) Scatter plot of the log‐transformed (log_10_) ratio of negative to positive interactions against degree for each taxon in different segments (duodenum, jejunum, ileum, and cecum). Red nodes: more negative interactions than positive interactions; blue nodes: more positive interactions than negative interactions.

We constructed microbial co‐occurrence networks for the four gut segments by integrating genus‐level interactions throughout the eight sampling time points (Supporting Information: Figure S9). The topological properties of the four networks were very different (Figure 5A and Supporting Information: Table S11). The vertex number (*n* = 274) and edge number (*n* = 19,626) of the network of the cecal microbial community were much larger than the other three gut segments. The clustering coefficient values of the networks for the jejunal microbiota (0.79) and the cecal microbiota (0.81) were higher than the other two, and the jejunal microbiota had lower values of average betweenness (36.0). The network for the duodenal and ileal microbiota exhibited higher values of average separation (1.74 and 1.79, respectively). These results suggested that the cecal microbiota exhibited a more complex microbial network with higher values of vertices, edges, clustering coefficient, and average betweenness and lower values of average separation. The first three gut segments possessed a relatively high proportion (from 52.1% to 92.8%) of generalist edges (present in at least two gut segments) but the cecum contained a much larger number (92.8%) of specialist edges (existing only in this gut segment) (Figure 5B), which suggests that many new interactions began building in the cecum with the increase in microbial abundance and diversity.

Next, we examined the keystone taxa, that is, the highly connected taxa, in the four co‐occurrence networks. The top 10 vertices with the most degrees in each network (34 genera in total) are shown in Figure 5C. *[Ruminococcus] torques group*, *Ruminiclostridium 9*, *Alternaria*/f_Nectriaceae, and *Ruminiclostridium 5* were the leading keystone taxa in the duodenum, jejunum, ileum, and cecum, respectively, and *Erysipelatoclostridium*, *Sellimonas*, *Butyricicoccus*, and *Ruminiclostridium 9* were keystone genera in more than one network. Among these 34 genera, 29 genera were in the “colonization” pattern, and four belonged to the “core” pattern, as we defined above (Supporting Information: Table S12). Notably, eight genera belonging to the family Ruminococcaceae were among the keystone taxa, which highlights their important roles in the chicken gut. We further analyzed the positive and negative associations of the genera in each network. Interestingly, an overwhelming majority of genera, including all of the keystone taxa, exhibited a higher proportion of positive associations than negative associations with other genera (Figure 5D). Only a few genera showed more negative relationships than positive relationships, including *Rhodotorula*, *Escherichia*, and *Shigella*.

### Serum metabolite abundance changes with chicken development

**Figure 6:**
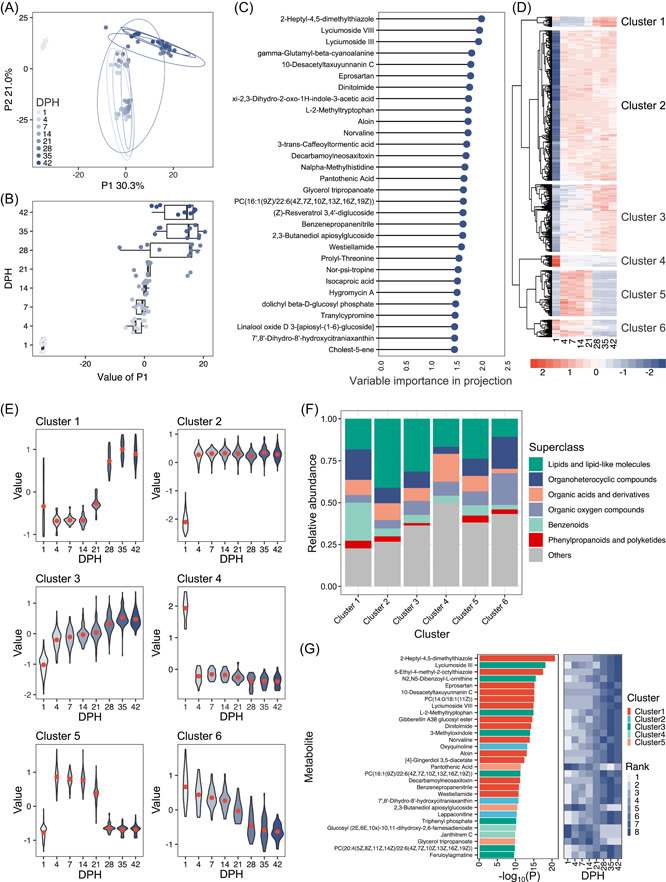
Changes in the metabolite profiles during chicken growth. (A) OPLS‐DA of metabolite profiles of the chicken serum at different time points. (B) Value of P1 generated from the OPLS‐DA at different time points. P1 explained 30.3% of the variation among the eight groups. (C) Importance of metabolites ranked by VIP. (D) Heatmap of metabolites significantly (GLM FDR < 0.05) altered over time. Metabolites are divided into six clusters based on hierarchical clustering. (E) Changes in metabolites of the six clusters over time. (F) Summary of pathway analysis for each cluster. (G) Top 30 most significantly different metabolites over time according to the results of GLM. The color of each bar represents the source of the cluster. The color of each cell reflects the rank of the abundance at different time points. GLM, general linear model; FDR, false discovery rate; OPLS‐DA, orthogonal partial least‐squares discriminant analysis; VIP, variable importance in projection.

We then performed time–course untargeted metabolomics to understand the alterations in the metabolites in the broiler serum during growth. First, orthogonal partial least‐squares discriminant analysis (OPLS‐DA) was used to cluster the samples, and three separated groups (birds at 1 DPH, from 4 to 21 DPH, and from 28 to 42 DPH) were found. The metabolic profile of the newly hatched birds (1 DPH) was very different from the other two growth stages (false discovery rate [FDR] < 0.05), and a shift occurred from 21 to 28 DPH (Figure 6A,B and Supporting Information: Table S13). To identify metabolites that significantly contributed to the group separation among different time points, we selected the metabolites with variable importance in projection (VIP) values greater than 1.5 as the cutoff (Figure 6C). A total of 30 metabolites met this criterion, and 2‐heptyl‐4,5‐dimethylthiazole, lycimumoside VIII, and lyciumoside III were the top three contributors to the group separation.

To identify the metabolites that changed over time, a general linear model (GLM) was performed. A total of 641 of the 1109 metabolites were associated with time (GLM, FDR < 0.05) and formed six different clusters (Figure 6D and Supporting Information: Table S14). Metabolites in these clusters followed different trajectories of abundance changes (Figure 6E). The abundance of metabolites in Cluster 1 (22 metabolites) and Cluster 3 (143 metabolites) increased over time, and the abundance of metabolites from Cluster 6 (37 metabolites) decreased gradually. Metabolites in Cluster 2 (318 metabolites) and Cluster 4 (24 metabolites) exhibited dramatic abundance changes after 1 DPH in exactly the opposite direction, from low to high and high to low, respectively. The abundance of metabolites in Cluster 5 (97 metabolites) first showed an increasing trend and then a decreasing trend. The six clusters differed in metabolite composition: Cluster 1 had a higher proportion of benzenoids, and Clusters 2 and 3 contained more lipid and lipid‐like molecules; organic acids and derivatives were more enriched in Cluster 4, and organic oxygen compounds were highly represented in Cluster 6 (Figure 6F). We analyzed the abundance changes of individual metabolites and found that 14 of the top 30 metabolites with the most variation in abundance were from Cluster 1, and all of them exhibited increased abundance over time (Figure 6G).

### QMP of gut microbiota establishes connections between microbes and chicken serum metabolites

**Figure 7:**
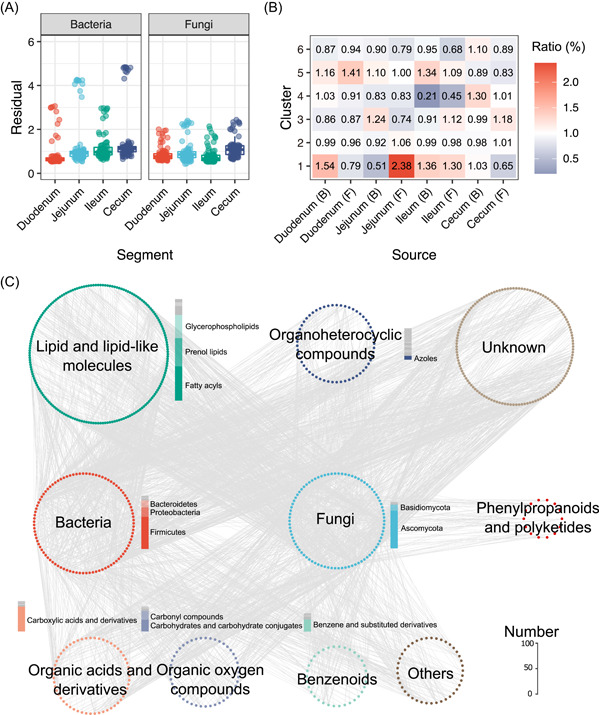
Microbe–metabolite interactions. (A) Residuals showing the difference in the microbe–metabolite association from different segments with absolute abundance. (B) Ratio of the top 1% co‐occurrence possibilities in all microbe–metabolite interactions. Red cells represent more interactions between the metabolite and the microbe; blue cells represent fewer interactions between the metabolite and the microbe. (C) Co‐occurrence network of the microbe–metabolite interactions discovered by mmvec from the four segments. Bar plots represent metabolite numbers in different subcategories. mmvec, microbiome–metabolite vectors.

To investigate the correlation between the dynamic changes in chicken gut microbes and their serum metabolites, we examined the explanation degree of the gut microbiota from different segments to the serum metabolic profile changes over time. We found that the bacterial and fungal communities from different gut segments were connected with the changes in the serum metabolic profile (*p* < 0.001, protest tests, Supporting Information: Table S15). A further comparison of the residuals in each test showed that residuals between the foregut microbiota and the serum metabolic profile were lower than the residuals between the hindgut microbiota and the serum metabolic profile (Figure 7A and Supporting Information: Table S16), which suggests that the diversity of microbiota in the chicken foregut (duodenum and jejunum) was low, but microbes in these gut segments may have more influence on the host metabolism. These results reinforced a segment effect on the strength of associations between microbial communities and serum metabolites.

We used microbiome–metabolite vectors (mmvec) to predict microbe–metabolite interactions based on their co‐occurrence probabilities in different gut segments. By selecting the top 1% microbe–metabolite interactions with the highest conditional probabilities, a co‐occurrence conditional probability heatmap for 180 genera from the four gut segments and 560 metabolites from the six clusters were generated according to mmvec (Supporting Information: Figure S10). We surveyed their distributions among the four gut segments and found that the gut microbiota was more associated with the abundance changes of metabolites in Clusters 1, 3, and 5 (Figure 7B). The jejunal fungi (2.38%) had the closest connections with metabolites in Cluster 1, followed by the duodenal bacteria (1.54%) and fungi (1.41%) with metabolites in Clusters 1 and 5, respectively. We also showed that the genera from the phyla Ascomycota and Firmicutes had closer connections with the metabolites in Clusters 2 and 3 for all four segments compared with the other phyla (Supporting Information: Table S17 and Figure S11).

To further explore the microbe–metabolite interactions, we constructed a network by integrating all possible microbe–metabolite interactions based on the interactions from the four segments (Figure 7C). We showed that more genera from the phyla Ascomycota (*n* = 68) and Firmicutes (*n* = 58) were associated with the metabolites, and the genera *Plectosphaerella*, *[Ruminococcus] torques group*, p__Ascomycota, *Butyricicoccus*, *Humicola*, o__Hyprcreales_2, and *Lactobacillus* were the top key taxa in the microbe–metabolite interactions (Figure 7C and Supporting Information: Table S18). The microbe‐associated metabolites were mainly from lipid and lipid‐like molecules (*n* = 184), followed by organoheterocyclic compounds (*n* = 57) and organic acids and derivatives (*n* = 55, Supporting Information: Table S19). Among the 184 microbe‐associated lipid and lipid‐like molecules, 62, 51, and 42 metabolites were from the classes of fatty acyls, prenol lipids, and glycerophospholipids, respectively, and more metabolites belonged to their subclasses of glycerophosphocholines (*n* = 28), fatty acid esters (*n* = 14), triterpenoids (*n* = 14), and terpene glycosides (*n* = 14, Supporting Information: Table S20). Metabolites in the category of organoheterocyclic compounds were dispersedly distributed at the class or subclass levels, with more compounds from azoles (*n* = 7), indoles and derivatives (*n* = 6), and pyridines and derivatives (*n* = 5). Most microbe‐associated organic acids and derivatives were from carboxylic acids and derivatives (*n* = 45), mainly the subclass amino acids, peptides, and analogs (*n* = 43), including essential amino acids (l‐lysine), nonessential amino acids (l‐tyrosine), nonproteinogenic amino acids (norvaline), and dipeptides (e.g., glutaminyl‐histidine and glutaminyl‐isoleucine).

We then predicted the sources of these metabolites using the MetOrigin platform. We identified 11 metabolites specifically derived from the gut microbiota, and 16 metabolites derived from both the host and gut microbiota, including l‐lysin, l‐tyrosine, and so on (Supporting Information: Table S21). The functional enrichment analysis indicated that these metabolites were mainly involved in amino acids metabolism (lysine degradation and valine, leucine, and isoleucine degradation) and lipid metabolism (sphingolipid metabolism and glycerophospholipid metabolism, Supporting Information: Table S22).

Taken together, QMP of the development of chicken gut microbiota established close connections between the gut microbes and the broiler serum metabolites involved in chicken metabolism.

## DISCUSSION

The current study investigated the developmental trajectories of the broiler chicken gut microbiota (bacteriome and mycobiome) using the QMP approach and connected the dynamic changes in the microbes in different gut segments with chicken serum metabolites during the 42‐day production period. To the best of our knowledge, this study is the first study to investigate the changes in bacteria and fungi in the chicken gut using a high‐throughput quantitative method. As evidenced in other microbiome studies, QMP has great advantages in minimizing the compositionality effects introduced by relative microbiome analyses [23, 26].

We found that bacteria and fungi rapidly colonized the chicken GIT during the first 7 days after birth. The early bacterial colonizers in the four gut segments are mainly aerobes and facultative anaerobes, such as *Streptococcus*, *Escherichia*, and *Shigella*. These bacteria are rapidly replaced by anaerobes even before 4 DPH, which is likely due to the rapid consumption of oxygen [27]. Although the diversity of the microbial communities reached a stable state at 7 DPH, the composition and function of the chicken gut microbiota may still change. For example, the expansion of the bacterial phylum Bacteroidetes, which is a complex carbohydrate degrader [28], began at 14 DPH in the cecum. Fungal community diversity was less variable with time and space. However, the high abundance of *Candida* in the jejunum and ileum from 4 DPH deserves more attention. Compared with bacteria and fungi, a low prevalence and abundance of archaea were found in the chicken gut. We also previously found that among 1978 species‐level metagenome‐assembled genomes from 799 chicken gut samples, only 8 species were archaeal species [24]. Litter and flies are potential sources for chicken gut archaea [29]. The low prevalence of archaea in the chicken gut may be due to modern farming systems with better hygiene conditions.

Previous studies showed that the microbial community composition in the human gut and natural environments revealed by QMP differed significantly from the composition revealed by RMP [21, 26, 30]. We also found inconsistency between the QMP and RMP of chicken gut microbiota in this study. First, QMP indicated that the absolute number of microbes in different chicken gut segments did not vary greatly, as revealed by RMP, and the AA was relatively stable over time compared with the RMP results (Figure 2A). This result suggested that RMP biased the real quantitative changes in the chicken gut microbiota. Second, RMP overestimated the microbial community structure variations and the influencing factors. Third, RMP missed many significant positive taxon–taxon interactions that were found in QMP, especially for the fungal community. These results suggest that quantitative approaches are necessary and will provide new clues to understand the changes in the chicken gut microbiota and the interactions between the microbes and the host.

Revealing the assembly and succession of the chicken gut microbiota during the production period may provide an opportunity for effective intervention to improve chicken health and production performance. Community type (or enterotype) is defined as a signature composition of gut microbes [31], the changes of which in longitudinal samples are frequently used to recognize gut microbial community variation [8, 9, 32]. By analyzing the community types, we demonstrated that the chicken gut microbiota, both bacteria and fungi, exhibited segment‐specific changes with the growth of the birds. Microbial source tracking analyses in this study and a previous study [27] indicated that a small proportion of microbes (10%–30%) in the posterior gut segments were from the adjacent anterior gut segments. The segment‐specific microbes in the chicken gut may be reasonably explained by the conditional colonization of microbes due to: (1) the selection pressures from the host, such as different physical structures, conditions, and functions in different gut segments; (2) the fitness and survival ability of the microbes; and (3) the competitive exclusion within the microbial community. This type of colonization may be a deterministic process from an ecological view. However, deterministic and stochastic processes jointly influence microbial community assembly [33]. We found that both processes controlled the colonization of microbes in the chicken gut. Deterministic processes dominated the bacterial community assembly, and stochastic processes governed the fungal community assembly. This difference may explain why the bacteriome exhibited more distinct community types and regular assembly processes than the mycobiome.

In addition to gut segment‐specific microbes, we also found stage‐specific taxa in the chicken gut. A stage‐specific microbial community was demonstrated to be critical to host development and health in humans and pigs [34, 35]. To identify the stage‐specific taxa, we classified the longitudinal occurrence patterns of the microbial community into three categories: “colonization,” “disappearance,” and “core.” Most bacterial and many fungal genera exhibited a colonization pattern, especially in the chicken cecum, including the well‐known beneficial bacterial genera *Lactobacillus*, *Bacteroides*, and Ruminococcaceae members and several rarely noticed fungal genera, such as *Tetracladium*, *Leohumicola*, *Pseudogymnoascus*, and *Mortierella*. Although more fungal genera belonged to the “disappearance” pattern than bacterial genera, which may have less competitive advantages, more fungal genera existed persistently (“core”) throughout the period. Among the 18 genera belonging to the “core” category, four fungi were present in all four gut segments. We also found close connections between fungi and chicken serum metabolites in subsequent analyses. These findings suggest a neglected role of the mycobiome in chicken development and health. The gut mycobiome has attracted increasing attention in recent microbiome studies [36]. For example, the gut mycobiome has close interactions with host immune cells, and disruption of commensal fungal communities influences local and peripheral immune responses and enhances relevant disease states in mice [37, 38].

Microbial co‐occurrence networks are widely used to investigate microbial relationships in gut microbiota, and the microbial interactions in a network are associated with distinct intensities of microbial competition or niche differentiation [39]. We showed that the chicken cecum hosted the most complex microbial networks compared with the small intestine, and the microbial interactions in the duodenum were more complicated than the interactions in the jejunum and ileum. Similar results were also found in the pig gut microbiota [40]. Although the absolute number of microbes in the duodenum was low, the microbial diversity was higher than in the jejunum and ileum, which may explain why there were more microbial interactions. We deduced that the higher microbial diversity in the duodenum was likely due to its cumulative reception of diverse microbes directly from the stomach. After subjection to selection pressures from the host, certain taxa expanded with increased numbers but fewer microbial interactions in the jejunum and ileum, and then the passengers entered the fermentation location and bloomed in the cecum.

Keystone taxa in a microbial interaction network are defined as highly connected taxa that may exert a considerable influence on microbiome structure functioning irrespective of their abundance [41]. We found that nearly all of the keystone taxa in the four networks had regular longitudinal occurrence patterns, that is, “colonization” and/or “core,” which suggests that the microbes with higher competitive advantages played a key role in stabilizing microbial interactions. All of the keystone taxa showed more positive associations than negative associations with other genera in the networks. The genus Ruminococcaceae is a typical representative. Eight genera from this family were keystone taxa with an occurrence pattern of “colonization.” Ruminococcaceae are common gut microbes in humans and animals that break down complex carbohydrates to produce beneficial products, such as short‐chain fatty acids, to maintain the functionality and morphology of intestinal epithelial cells and regulate gut microbiota balance. Ruminococcaceae species have close interactions with other microbes via cross‐feeding [42]. Our results confirmed the role of Ruminococcaceae bacteria as keystone taxa and their positive correlations with other microbes in the chicken gut. However, there are research gaps in our understanding of the function of this family in chicken health, development, and production performance, which deserve more attention in the future.

We found that although microbes, especially the fungi in the foregut, had low abundance, they had close connections with chicken serum metabolites. Associations between the foregut microbiota and chicken metabolism and growth were also observed in previous studies [6, 43]. This result may be due to the direct absorption of microbial metabolites or an indirect influence of microbes on dietary nutrient absorption in the small intestine. Dysbiosis of the microbiota in the small intestine in humans is implicated in functional gastrointestinal disorders and chronic liver disease [44, 45]. Therefore, more efforts should be made to reveal the roles and underlying mechanisms of the foregut microbiota in chicken growth and development in the future.

The gut microbiota is an important determinant of the host serum metabolic landscape [46, 47]. We showed that more correlations existed between microbes from the phyla Ascomycota and Firmicutes and the serum metabolites (Figure 7C). Ascomycota and Firmicutes are the dominant phyla in the chicken intestinal fungal and bacterial communities, respectively [19, 48], and our results confirmed their critical impact on chicken physiology and metabolism, especially lipid and amino acid metabolism.

Accumulating evidence has shown that microbes alter circulating lipid concentrations and affect lipid homeostasis in the host [49]. We found that the chicken gut microbiota was mostly associated with compounds belonging to fatty acyls, prenol lipids, and glycerophospholipids. For the class of fatty acyls, arachidonic acid was associated with the greatest number of gut microbes. This result is also supported by previous results showing that an altered microbial community significantly disturbs arachidonic acid metabolism [50]. Arachidonic acid may be metabolized to generate an impressive spectrum of biologically active fatty acid mediators and is a key substance related to chicken meat flavor [51, 52]. For glycerophospholipids, 28 metabolites belonging to glycerophosphocholines were related to the chicken gut microbiota, which is similar to the results in mice that gut microbiota change the mouse lipid signature by altering the acyl‐chain profile of glycerophospholipids, including phosphatidylcholine, ethanolamine, and inositol [53]. We also found that host genotype‐dependent microbes were highly associated with chicken muscle lipid metabolism in a previous study [54].

Gut microbiota de novo synthesize essential amino acids that are implicated in amino acid homeostasis in the host [55]. We found that the level of l‐lysine, which is the second limiting amino acid in broiler corn‐soybean meal‐based diets [56], was closely associated with the gut microbiota in the chicken serum. This result is consistent with a previous finding that approximately 75% of the total l‐lysine produced by the gut microbiota was absorbed in the pig small intestine [57]. We also showed that l‐tyrosine, a non‐essential amino acid that is higher in the GIT than in other tissues [58], was associated with the gut microbiota. We also demonstrated connections between the gut microbiota and peptides and several amino acid analogs, such as gabapentin and l‐norvaline. Dipeptides play important roles in protecting the intestinal environment against oxidative stress and modulating the immune system [59, 60, 61]. Gabapentin is a synthetic analog of gamma‐aminobutyric acid that improves growth performance by increasing body weight and feed intake [62]. l‐norvaline promotes tissue regeneration and muscle growth [63].

## CONCLUSION

In summary, we analyzed the development of the broiler gut bacteriome and mycobiome based on the QMP strategy and revealed potential connections between the gut microbiota and chicken serum metabolites. We showed that RMP biased chicken gut microbiota development and microbial interactions and revealed the segment‐ and stage‐specific features of chicken gut microbiota assembly using the QMP method. We also highlighted the importance of the foregut microbiota and the mycobiome in chicken lipid and amino acid metabolism. Our study presented a comprehensive view of the dynamic changes in gut microbiota during chicken growth and provided further insights into the influence of gut microbes on chicken metabolism. We should stress that the connections we observed between the gut microbiota and chicken serum metabolites are just based on association analysis; future experimental validation efforts are necessary for confirming our findings.

## METHODS

### Animals and sample collection

Two hundred male chicks (Arbor Acres) that hatched on the same day were reared on the same poultry farm (Zhuozhou, China). The diet formula of broilers was formulated according to the feeding standards of Chinese chickens (NY/T33‐2004; Supporting Information: Table S1). The broilers were divided into 10 flocks. The flocks were sampled eight times according to the study design, and one bird per flock was randomly selected each time. Whole blood and the luminal content of four gut segments (the duodenum, jejunum, ileum, and cecum) were collected. More details for animals and sample collection are included in the Supporting Information: Methods.

### DNA extraction and quantification

Microbial DNA extraction was performed according to the protocol described previously [64]. Because mechanical lysis and repeated head beating could increase the DNA extraction efficiency [65], we performed three successive rounds of bead beating with glass beads. Microbial genomic DNA from the luminal contents of each segment was extracted using the QIAamp DNA Stool Mini Kit (catalog no. 51504; Qiagen). The weight of the samples was recorded during the process of DNA extraction (Supporting Information: Table S2).

For microbial enumeration, we first constructed a 10‐log‐fold standard curve that ranged from 10^3^ to 10^8^ copies using appropriate reference organisms with known copy numbers of 16S or internal transcribed spacer (ITS) rRNA genes (*Escherichia coli* for total bacteria and archaea, *Komagataella pastoris* for fungi) as described previously [66].

Quantification of total bacteria, archaea, and fungi was carried out by quantitative PCR using an ABI 7500 Real‐time PCR system (Applied Biosystems) with a 20 μL reaction volume containing 10 μL TB Green® Premix Ex Taq™ (RR420A; Takara). The primers used for the quantification of total bacteria were 5′‐TCCTACGGGAGGCAGCAGT‐3′ (forward) and 5′‐GGACTACCAGGGTATCTAATCCTGTT‐3′ (reverse) [67]. The primers used for the quantification of total fungi were 5′‐CTTGGTCATTTAGAGGAAGTAA‐3′ (forward) and 5′‐TCCTCCGCTTATTGATATGC‐3′ (reverse) [68]. More details for DNA extraction and quantification of the gut microbiota are included in the Supporting Information: Methods.

### Amplicon sequencing and data processing

To run domain‐specific PCRs, samples were split into two aliquots, and each aliquot was amplified with a specific primer pair: the primers V3–V4 (F: 5′‐CCTACGGGNBGCASCAG‐3′ and R: 5′‐GACTACNVGGGTATCTAATCC‐3′) for prokaryotes (bacteria and archaea) [69] or the primers ITS2 (F: 5′‐GTGARTCATCGAATCTTT‐3′ and R: 5′‐GATATGCTTAAGTTCAGCGGGT‐3′) for eukaryotes [70]. The pooled library was sequenced on the Illumina HiSeq platform (2 × 250 bp, Supporting Information: Table S3).

Raw fastq files were quality‐filtered and taxonomically analyzed using QIIME2 (v2019.7) [71]. We assembled quality‐filtered reads into amplicon sequence variants using DADA2 (v1.10.0) [72]. Taxonomy assignment was performed on the basis of the SILVA database (v132) [73] and UNITE database (v8) [74]. The Shannon index and principal coordinate analysis (PCoA) based on Bray–Curtis dissimilarity were calculated using the R package vegan [75]. Statistical analyses for alpha diversity and beta diversity were performed using the Kruskal–Wallis test/Wilcoxon rank‐sum test and permutational multivariate analysis of variance (PERMANOVA) [76], respectively. The symmetric Procrustes correlation coefficients between the microbiome based on RA and AA and *p* values were obtained using the “protest” function in vegan. More details for data procession are included in the Supporting Information: Methods.

### Enterotype clustering and the stochasticity of the gut microbiota assembly

DMM models were used to assign the samples to the community types [77], and bin samples based on the log‐transformed AA. The FEAST (v1.0.0) algorithm was used to track the sources of microbial populations [78]. Each sampling site was identified as a sink, starting with the jejunum, and all other segments were treated as potential sources. Only the proportion of microbes sourced from the adjacent anterior gut segment was considered in the present study. To assess the relative importance of determinism and stochasticity in microbiome assembly, a two‐step procedure was applied considering β‐nearest taxon index (βNTI) and Bray–Curtis‐based Raup–Crick Index (RC_Bray_) values, including heterogeneous selection, homogeneous selection, dispersal limitation, homogenizing dispersal, and undominated [25]. The stochastic process includes three processes (homogenizing dispersal, dispersal limitation, and undominated), and the deterministic processes include two processes (heterogeneous selection and homogeneous selection). The values βNTI and RC_Bray_ were calculated using the R package iCAMP (v1.3.4) [79]. More details for enterotype clustering and the stochasticity of the gut microbiota assembly are included in the Supporting Information: Methods.

### Microbial co‐occurrence network analysis

Microbial taxon–taxon co‐occurrence networks were constructed using partial Spearman correlation using the R package ppcor (v1.1) in different segments. To reduce noise and false‐positive predictions, network inclusion was restricted to genera that were present in at least 30% of samples. The FDR was used in each microbial network, and correlations with adjusted *p* values above 0.05 were filtered out [80]. Topological features were estimated using the R package igraph (v1.2.11) [81]. Edges present in only one subnetwork were specialist edges, and edges in more than one subnetwork were considered generalist edges. Keystone taxa are highly connected taxa that exert a considerable influence on microbiome structure and functioning [41]. More details for microbial co‐occurrence network analysis are included in the Supporting Information: Methods.

### Metabolic profiling of chicken serum and data processing

We performed untargeted metabolomics to analyze the metabolites in chicken serum. To analyze the metabolites in chicken serum, an Agilent 1200 series high‐performance liquid chromatography system (Agilent Technologies) was used to separate the metabolites. Agilent Mass Hunter workstation software was used for data analyses and compound identification. The downstream analyses were based on the annotations generated from the ME‐Tabolite LINk (METLIN) [82] and Human Metabolome Database (HMDB) [83] databases. We filtered the features in two steps: (1) we retained the metabolic features present in >30% of the samples and (2) we filtered out the unknown metabolic features according to the annotations from the databases. A total of 1109 different kinds of metabolites were used for downstream analyses.

The metabolites of serum at different time points were assessed using OPLS‐DA in the R package ropls (v1.24.0) [84]. The VIP was calculated, which reflects the loading weight and the variability of the response explained by this component. We clustered the metabolites into six clusters using the R package pheatmap (v1.0.12). Pathway analysis of metabolites in each cluster was performed using MetaboAnalyst 5.0 (http://www.metaboanalyst.ca) [85]. The category and functional enrichment analyses of the associated metabolites were analyzed using the MetOrigin platform [86]. More details for metabolic profiling and data processing are included in the Supporting Information: Methods.

### Microbe–metabolite interactions

To examine the association between the microbiota and the metabolome, we analyzed the symmetric Procrustes correlation coefficients between the microbiome based on relative/AA and metabolic profiling. *P* values were obtained using the “protest” function in vegan (v2.6‐4). The residuals of the correlations were generated from the results of the “procrustes” function from the R package vegan. Microbiota from different segments and metabolome feature tables were analyzed using mmvec [87] to identify microbe–metabolite interactions based on their co‐occurrence probabilities as predicted by neural networking. The top 1% microbe–metabolite interactions with the highest conditional probabilities were regarded as positive co‐occurrences.

### Statistical analysis and visualization

The correlations not mentioned above were calculated using the R function “cor.test”. When multiple hypotheses were considered simultaneously, *P* values were adjusted to control the FDR [80]. The intersecting sets were analyzed using the R package UpSetR (v1.4.0) [88]. The co‐occurrence networks were visualized using Gephi (v0.9.2, https://gephi.org/). The heatmap of microbe–metabolite interactions was visualized using the R package ComplexHeatmap (v2.8.0) [89]. Most of the figures in this study were visualized using the R packages ggplot2 (v3.3.5) and patchwork (v1.1.1). The significantly changed metabolites during chicken growth were examined using GLMs on time series metabolic data.

## AUTHOR CONTRIBUTIONS

Yongfei Hu and Yuming Guo designed the study. Yuqing Feng performed all bioinformatics and statistical analyses; Yuqing Feng, Meihong Zhang, Yan Liu, Xinyue Yang, and Fuxiao Wei performed the experiments. Yuqing Feng interpreted the data and wrote the manuscript. All of the other authors revised and edited the manuscript. All authors read and approved the final manuscript.

## CONFLICT OF INTEREST STATEMENT

The authors declare no conflict of interest.

## ETHICS STATEMENT

The ethics application (No. AW301122002‐1‐1) was approved by the Animal Welfare Committee of China Agricultural University.

## Supporting information

Supporting information.

Supporting information.

## Data Availability

All the sequencing data have been deposited in NCBI Sequence Read Archive (SRA) under accession number PRJNA817429 (https://www.ncbi.nlm.nih.gov/sra/?term=PRJNA817429) and the National Microbiology Data Center accession number NMDC10018360 (https://nmdc.cn/resource/genomics/project/detail/NMDC10018360). The scripts used are saved in GitHub https://github.com/yuqing-feng/scripts_for_iMeta_2023_11.git. Supporting Information: Materials (figures, tables, graphical abstract, slides, videos, Chinese translated version, and updated materials) may be found in the iMeta Science http://www.imeta.science/.
