# Supplementary material for: Quantitative microbiome profiling reveals the developmental trajectory of the chicken gut microbiota and its connection to host metabolism
Source: Imeta. 2023 Apr 25;2(2):e105. doi: 10.1002/imt2.105 (PMC10989779; doi:10.1002/imt2.105)
Supplement: Supplementary file 1 — Supporting information. [file IMT2-2-e105-s001.pdf]

Supporting Information for

Quantitative microbiome profiling reveals the developmental trajectory of the chicken gut microbiota and its connection to host metabolism

Running title: Developmental trajectory of the chicken gut microbiota

Yuqing Feng, Meihong Zhang, Yan Liu, Xinyue Yang, Fuxiao Wei, Xiaolu Jin, Dan Liu, Yuming Guo*, Yongfei Hu*

State Key Laboratory of Animal Nutrition, College of Animal Science and Technology, China Agricultural University, Beijing 100193, China

***Correspondence**

huyongfei@cau.edu.cn (Yongfei Hu)

guoyum@cau.edu.cn (Yuming Guo)

Methods

Animals and sample collection

Two hundred male chicks (Arbor Acres) that hatched on the same day were reared on the same poultry farm (Zhuozhou, China) with free access to water and corn-soybean-based diets. The diet formula of broilers was formulated according to the feeding standards of Chinese chickens (NY/T33-2004, Supplementary Table S1), and contained 3.0 kcal/kg metabolizable energy and 21.5% crude protein. The broilers were divided into 10 flocks (20 birds per flock). The birds were kept at ambient temperature of 20°C, relative humidity of 50% and a lighting program of 16L/8D. The flocks were sampled eight times (1, 4, 7, 14, 21, 28 35 and 42 DPH) according to the study design, and one bird per flock was randomly selected each time. Whole blood of the selected birds was collected from the wing vein, and serum samples were obtained by centrifuging at 4000 × g for 15 min, which was then promptly frozen at -20°C for further analyses. The luminal content of four gut segments (the duodenum, jejunum, ileum, and cecum) was collected and frozen in liquid nitrogen. All of the samples were transported to the laboratory and stored at -80°C for further analyses.

DNA extraction and quantification

Microbial DNA extraction was performed according to the protocol described previously [1]. Because mechanical lysis and repeated head beating could increase the DNA extraction efficiency [2], we performed three successive rounds of bead beating with glass beads (1 min, 30 s, and 30 s). Microbial genomic DNA from the luminal contents of each segment was extracted using the QIAamp DNA Stool Mini Kit (catalog no. 51504, Qiagen, Hilden, Germany). The weight of the samples was recorded during the process of DNA extraction (Supplementary Table S2).

For microbial enumeration, we first constructed a 10-log-fold standard curve that ranged from 10^3 to 10^8 copies using appropriate reference organisms with known copy numbers of 16S or internal transcribed spacer (ITS) rRNA genes (*Escherichia coli* for total bacteria and archaea, *Komagataella pastoris* for fungi) as described previously [3]. The genomes of *E. coli* and *K. pastoris* were extracted using a TIANamp Bacteria DNA Kit (DP302, Tiangen, Beijing, China) and a TIANamp Yeast DNA Kit (DP307, Tiangen, Beijing, China), respectively. The DNA concentration of the extracted microbial

genomes was quantified by a Qubit® fluorometer (Invitrogen, Carlsbad, CA, USA). The number of bacteria and fungi was calculated per ng of DNA using an online calculator (https://cels.uri.edu/gsc/cndna.html), according to the following Euqation (1):

$$\text{Number of copies} = (\text{Amount} \times 6.022 \times 10^{23}) / (\text{Length} \times 1 \times 10^{9} \times 650) \quad (1)$$

Quantification of total bacteria, archaea, and fungi was carried out by quantitative PCR using an ABI 7500 Real-time PCR system (Applied Biosystems, Waltham, MA, USA) with a 20-μL reaction volume containing 10 μL TB Green® Premix Ex Taq™ (RR420A, Takara, Dalian, China). The primers used in this study were synthesized by Sango Biotech Co., Ltd. (Shanghai, China). The primers used for the quantification of total bacteria were 5'-TCCTACGGGAGGCAGCAGT-3' (forward) and 5'-GGACTACCAGGGTATCTAATCCTGTT-3' (reverse) [4]. The primers used for the quantification of total fungi were 5'-CTTGGTCATTTAGAGGAAGTAA-3' (forward) and 5'-TCCTCCGCTTATTGATATGC-3' (reverse) [5]. The absolute abundance of taxon A was calculated by Equation (2):

$$\text{Estimated absolute abundance of taxon A} = \text{Relative abundance of taxon A in bacteria/archaea/fungi} \times \text{Total number of gene copies of bacteria/archaea/fungi} \quad (2)$$

The relative abundance of taxon A in bacteria or archaea was calculated from the following amplicon sequencing data of the V3-V4 regions of the 16S rRNA gene and from the ITS2 region in fungi.

Amplicon sequencing and data processing

To run domain-specific PCRs, samples were split into two aliquots, and each aliquot was amplified with a specific primer pair: the primers V3-V4 (F: 5'-CCTACGGGNBGCASCAG-3' and R: 5'-GACTACNVGGGTATCTAATCC-3') for prokaryotes (bacteria and archaea) [6] or the primers ITS2 (F: 5'-GTGARTCATCGAATCTTT-3' and R: 5'-GATATGCTTAAGTTCAGCGGGT-3') for

eukaryotes [7]. The pooled library was sequenced on the Illumina HiSeq platform (2 × 250 bp, Supplementary Table S3).

Raw fastq files were quality-filtered and taxonomically analyzed using QIIME2 (v2019.7) [8]. We assembled quality-filtered reads into amplicon sequence variants (ASVs) using DADA2 (v1.10.0) [9]. ASVs were filtered out when they were present in fewer than two samples or had a feature count of less than 10. Taxonomy assignment was performed using a pretrained naïve Bayes classifier on the basis of the SILVA database (v132) [10] and UNITE database (v8) [11]. For taxonomic annotation, all unassigned sequences and sequences annotated as mitochondria and chloroplasts were removed. Samples were rarefied to the same number of reads for the downstream analyses. The Shannon index and principal coordinate analysis (PCoA) based on Bray-Curtis dissimilarity were calculated using the R package vegan [12]. Statistical analyses for alpha diversity and beta diversity were performed using the Kruskal-Wallis test/Wilcoxon rank-sum test and permutational multivariate analysis of variance (PERMANOVA) [13], respectively. The symmetric Procrustes correlation coefficients between the microbiome based on relative abundance and absolute abundance and P values were obtained using the "protest" function in vegan.

Enterotype clustering and the stochasticity of the gut microbiota assembly

Dirichlet multinomial mixture (DMM) models were used to assign the samples to the community types [14], and bin samples based on the log-transformed absolute abundance. The appropriate number of clusters was determined based on the lowest Laplace approximation score.

The fast expectation-maximization microbial source tracking (FEAST, v1.0.0) algorithm was used to track the sources of microbial populations [15]. FEAST is a highly efficient expectation-maximization-based method that estimates the fraction of a microbial community contributed by a potential source environment. Each sampling site was identified as a sink, starting with the jejunum, and all other segments were treated as potential sources. Only the proportion of microbes sourced from the adjacent anterior gut segment was considered in the present study.

To assess the relative importance of determinism and stochasticity in microbiome assembly, a two-step procedure was applied considering β-nearest taxon index (βNTI) and Bray-Curtis-based Raup-

Crick Index (RC_{Bray}) values, including heterogeneous selection ($\beta NTI < -2$), homogeneous selection ($\beta NTI > 2$), dispersal limitation ($|\beta NTI| < 2$ and $RC_{Bray} > 0.95$), homogenizing dispersal ($|\beta NTI| < 2$ and $RC_{Bray} < -0.95$), and undominated ($|\beta NTI| < 2$ and $|RC_{Bray}| < 0.95$) [16]. The stochastic process includes three processes (homogenizing dispersal, dispersal limitation, and undominated), and the deterministic processes include two processes (heterogeneous selection and homogeneous selection). The values βNTI and RC_{Bray} were calculated using the R package iCAMP (v1.3.4) [17].

Microbial cooccurrence network analysis

Microbial taxon-taxon cooccurrence networks were constructed using partial Spearman correlation using the R package ppcor (v1.1) in different segments. To reduce noise and false-positive predictions, network inclusion was restricted to genera that were present in at least 30% of samples. The false discovery rate (FDR) was used in each microbial network, and correlations with adjusted P values above 0.05 were filtered out [18]. Topological features were estimated using the R package igraph (v1.2.11) [19]. The edge number, vertices number, clustering coefficient, mean betweenness centrality, and average separation were calculated with functions embedded in the R package igraph. Edges present in only one subnetwork were specialist edges, and edges in more than one subnetwork were considered generalist edges. Keystone taxa are highly connected taxa that exert a considerable influence on microbiome structure and functioning [20]. We identified the genera at the top degree from the four subnetworks as keystone taxa.

Metabolic profiling of chicken serum and data processing

We performed untargeted metabolomics to analyze the metabolites in chicken serum. The frozen serum samples were melted and mixed with 400 μL of a methanol/acetonitrile (1:1, v/v) mixture. The mixture was vortexed for 30 s, followed by ultrasonic extraction at 4°C for 10 min and incubation at -20°C for 60 min. After centrifugation at 12,000 × g for 15 min, the residue was removed, and the supernatant was collected. The supernatant was dried under nitrogen. The dried extractions were redissolved in 100 μL of a methanol/acetonitrile (1:1, v/v) mixture. The mixture was ultrasonically extracted at 4°C for 10 min. The supernatant was collected after centrifugation at 12,000 × g for 15 min.

To analyze the metabolites in chicken serum, an Agilent 1200 series high performance liquid chromatography (HPLC) system (Agilent Technologies, Santa Clara, CA, USA) equipped with an ACQUITY ultraperformance LC (UPLC) BEH C18 column (100 mm × 2.1 mm i.d., 1.7 μm, Waters, Milford, MA, USA) was used to separate the metabolites. For the positive mode, mobile phases A and B were water (containing 0.1% formic acid) and acetonitrile (containing 0.1% formic acid), respectively. For the negative mode, mobile phases A and B were water (containing 5 mM ammonium formate) and acetonitrile (containing 5 mM ammonium formate), respectively. The following gradient was used: 0 min, 15% B; 2 min, 15% B; 10 min, 30% B; 30 min, 90% B; 35 min, 90% B; 36 min, 15% B; and 45 min, 15% B. The flow rate was 400 μL/min, the injection volume was 3 μL, and the column temperature was 45°C. An Agilent 6510 ESI-Q-TOF system from Agilent Technologies was used for mass spectral (MS) acquisition. The positive and negative ionization modes were used. Agilent Mass Hunter workstation software was used for data analyses and compound identification. The downstream analyses were based on the annotations generated from the METLIN [21] and HMDB [22] databases. We filtered the features in two steps: 1) we retained the metabolic features present in > 30% of the samples; and 2) we filtered out the unknown metabolic features according to the annotations from the databases. A total of 1,109 different kinds of metabolites were used for downstream analyses.

The metabolites of serum at different time points were assessed using orthogonal partial least-squares discriminant analysis (OPLS-DA) in the R package ropls [23]. The variable importance in projection (VIP) was calculated, which reflects the loading weight and the variability of the response explained by this component. We clustered the metabolites into six clusters using the R package pheatmap (v1.0.12). Pathway analysis of metabolites in each cluster was performed using MetaboAnalyst 5.0 (http://www.metaboanalyst.ca) [24]. The category and functional enrichment analysis of the metabolites were analyzed using the MetOrigin [25].

Microbe-metabolite interactions

To examine the association between the microbiota and the metabolome, we analyzed the symmetric Procrustes correlation coefficients between the microbiome based on relative/absolute abundance and

metabolic profiling. *P* values were obtained using the "protest" function in vegan. The residuals of the correlations were generated from the results of the "procrustes" function from the R package vegan. Microbiota from different segments and metabolome feature tables were analyzed using microbiome-metabolite vectors (mmvec) [26] to identify microbe-metabolite interactions based on their co-occurrence probabilities as predicted by neural networking. The top 1% microbe-metabolite interactions with the highest conditional probabilities were regarded as positive co-occurrences.

Statistical analysis and visualization

The correlations not mentioned above were calculated using Spearman's correlations using the R function cor.test. When multiple hypotheses were considered simultaneously, *P* values were adjusted to control the FDR [18]. The intersecting sets were analyzed using the R package UpSetR (v1.4.0) [27]. The co-occurrence networks were visualized using Gephi (v0.9.2) [28]. The heatmap of microbe-metabolite interactions was visualized using the R package ComplexHeatmap (v2.8.0) [29]. Most of the figures in this study were visualized using the R packages ggplot2 (v3.3.5) and patchwork (v1.1.1). The significantly changed metabolites during chicken growth were examined using general linear models (GLMs) on time series metabolic data.

References

1. Jian, Ching, Panu Luukkonen, Hannele Yki-Jarvinen, Anne Salonen, Katri Korpela. 2020. "Quantitative PCR provides a simple and accessible method for quantitative microbiota profiling." *PLoS One* 15: e0227285. https://doi.org/10.1371/journal.pone.0227285

2. Costea, Paul I, Georg Zeller, Shinichi Sunagawa, Eric Pelletier, Adriana Alberti, Florence Levenez, Melanie Tramontano, et al. 2017. "Towards standards for human fecal sample processing in metagenomic studies." *Nature Biotechnology* 35: 1069-1076. https://doi.org/10.1038/nbt.3960

3. Rinttila, T., A. Kassinen, E. Malinen, L. Krogius, A. Palva. 2004. "Development of an extensive set of 16S rDNA-targeted primers for quantification of pathogenic and indigenous bacteria in faecal samples by real-time PCR." *Journal of Applied Microbiology* 97: 1166-1177. https://doi.org/10.1111/j.1365-2672.2004.02409.x

4. Nadkarni, Mangala A., F. Elizabeth Martin, Nicholas A. Jacques, Neil Hunter. 2002. "Determination of bacterial load by real-time PCR using a broad-range (universal) probe and primers set." *Microbiology (Reading)* 148: 257-266. https://doi.org/10.1099/00221287-148-1-257

5. Manter, Daniel K., Jorge M. Vivanco. 2007. "Use of the ITS primers, ITS1F and ITS4, to characterize fungal abundance and diversity in mixed-template samples by qPCR and length heterogeneity analysis." *Journal of Microbiological Methods* 71: 7-14. https://doi.org/10.1016/j.mimet.2007.06.016

6. Takahashi, Shunsuke, Junko Tomita, Kaori Nishioka, Takayoshi Hisada, Miyuki Nishijima. 2014. "Development of a prokaryotic universal primer for simultaneous analysis of bacteria and archaea using next-generation sequencing." *PLoS One* 9: e105592. https://doi.org/10.1371/journal.pone.0105592

7. Lemoinne, Sara, Astrid Kemgang, Karima Ben Belkacem, Marjolene Straube, Sarah Jegou, Christophe Corpechot, Olivier Chazouilleres, Chantal Housset, Harry Sokol. 2020. "Fungi participate in the dysbiosis of gut microbiota in patients with primary sclerosing cholangitis." *Gut* 69: 92-102. https://doi.org/10.1136/gutjnl-2018-317791

8. Bolyen, Evan, Jai Ram Rideout, Matthew R. Dillon, Nicholas A. Bokulich, Christian C. Abnet, Gabriel A. Al-Ghalith, Harriet Alexander, et al. 2019. "Reproducible, interactive, scalable and extensible microbiome data science using QIIME 2." *Nature Biotechnology* 37: 852-857. https://doi.org/10.1038/s41587-019-0209-9

9. Callahan, Benjamin J., Paul J. McMurdie, Michael J. Rosen, Andrew W. Han, Amy Jo A. Johnson, Susan P. Holmes. 2016. "DADA2: high-resolution sample inference from illumina amplicon data." *Nature Methods* 13: 581-583. https://doi.org/10.1038/nmeth.3869

10. Quast, Christian, Elmar Pruesse, Pelin Yilmaz, Jan Gerken, Timmy Schweer, Pablo Yarza, Jörg Peplies, Frank Oliver Glockner. 2013. "The SILVA ribosomal RNA gene database project: Improved data processing and web-based tools." *Nucleic Acids Research* 41: D590-596. https://doi.org/10.1093/nar/gks1219

11. Nilsson, Rolf Henrik, Karl-Henrik Larsson, Andy F S Taylor, Johan Bengtsson-Palme, Thomas S Jeppesen, Dmitry Schigel, Peter Kennedy, et al. 2019. "The UNITE database for molecular identification of fungi: Handling dark taxa and parallel taxonomic classifications." *Nucleic Acids Research* 47: D259-D264. https://doi.org/10.1093/nar/gky1022

12. Dixon, Philip. 2003. “VEGAN, a package of R functions for community ecology.” *Journal of Vegetation Science* 14: 927-930. https://doi.org/10.1111/j.1654-1103.2003.tb02228.x

13. Zapala, Matthew A., Nicholas J. Schork. 2006. “Multivariate regression analysis of distance matrices for testing associations between gene expression patterns and related variables.” *Proceedings of the National Academy of Sciences of the United States of America* 103: 19430-19435. https://doi.org/10.1073/pnas.0609333103

14. Holmes, Ian, Keith Harris, Christopher Quince. 2012. “Dirichlet multinomial mixtures: Generative models for microbial metagenomics.” *PLoS One* 7: e30126. https://doi.org/10.1371/journal.pone.0030126

15. Shenhav, Liat, Mike Thompson, Tyler A Joseph, Leah Briscoe, Ori Furman, David Bogumil, Itzhak Mizrahi, Itsik Pe'er, Eran Halperin. 2019. “FEAST: Fast expectation-maximization for microbial source tracking.” *Nature Methods* 16: 627-632. https://doi.org/10.1038/s41592-019-0431-x

16. Xiong, Chao, Brajesh K Singh, Ji-Zheng He, Yan-Lai Han, Pei-Pei Li, Li-Hua Wan, Guo-Zhong Meng, et al. 2021. “Plant developmental stage drives the differentiation in ecological role of the maize microbiome.” *Microbiome* 9: 171. https://doi.org/10.1186/s40168-021-01118-6

17. Ning, Daliang, Mengting Yuan, Linwei Wu, Ya Zhang, Xue Guo, Xishu Zhou, Yunfeng Yang, Adam P Arkin, Mary K Firestone, Jizhong Zhou. 2020. “A quantitative framework reveals ecological drivers of grassland microbial community assembly in response to warming.” *Nature Communications* 11: 4717. https://doi.org/10.1038/s41467-020-18560-z

18. Benjamini, Yoav, Yosef Hochberg. 1995. “Controlling the false discovery rate - a practical and powerful approach to multiple testing.” *Journal of the Royal Statistical Society Series B-Statistical Methodology* 57: 289-300. https://doi.org/10.1111/j.2517-6161.1995.tb02031.x

19. Csárdi, Gábor, Tamás Nepusz. 2006. “The igraph software package for complex network research.” *International Journal of Complex Systems* 1695.

20. Banerjee, Samiran, Klaus Schlaeppi, Marcel G. A. van der Heijden. 2018. “Keystone taxa as drivers of microbiome structure and functioning.” *Nature Reviews Microbiology* 16: 567-576. https://doi.org/10.1038/s41579-018-0024-1

21. Guijas, Carlos, J. Rafael Montenegro-Burke, Xavier Domingo-Almenara, Amelia Palermo, Benedikt Warth, Gerrit Hermann, Gunda Koellensperger, et al. 2018. “METLIN: A technology platform for identifying knowns and unknowns.” *Analytical Chemistry* 90: 3156-3164. https://doi.org/10.1021/acs.analchem.7b04424

22. Wishart, David S, Yannick Djoumbou Feunang, Ana Marcu, An Chi Guo, Kevin Liang, Rosa Vazquez-Fresno, Tanvir Sajed, et al. 2018. “HMDB 4.0: The human metabolome database for 2018.” *Nucleic Acids Research* 46: D608-D617. https://doi.org/10.1093/nar/gkx1089

23. Thevenot, Etienne A, Aurelie Roux, Ying Xu, Eric Ezan, Christophe Junot. 2015. “Analysis of the human adult urinary metabolome variations with age, body mass index, and gender by implementing a comprehensive workflow for univariate and OPLS statistical analyses.” *Journal of Proteome Research* 14: 3322-3335. https://doi.org/10.1021/acs.jproteome.5b00354

24. Pang, Zhiqiang, Jasmine Chong, Guangyan Zhou, David Anderson de Morais, Le Chang, Michel Barrette, Carol Gauthier, Pierre-Étienne Jacques, Shuzhao Li, Jianguo Xia. 2021. “MetaboAnalyst 5.0: Narrowing the gap between raw spectra and functional insights.” *Nucleic Acids Research* 49: W388-W396. https://doi.org/10.1093/nar/gkab382

25. Yu, Gang, Cuifang Xu, Danni Zhang, Feng Ju, Yan Ni. 2022. “MetOrigin: discriminating the origins of microbial metabolites for integrative analysis of the gut microbiome and metabolome.” *iMeta* 1: e10. https://doi.org/10.1002/imt2.10

26. Morton, James T, Alexander A Aksenov, Louis Felix Nothias, James R Foulds, Robert A Quinn, Michelle H Badri, Tami L Swenson, et al. 2019. “Learning representations of microbe-metabolite interactions.” *Nature Methods* 16: 1306-1314. https://doi.org/10.1038/s41592-019-0616-3

27. Conway, Jake R., Alexander Lex, Nils Gehlenborg. 2017. “UpSetR: An R package for the visualization of intersecting sets and their properties.” *Bioinformatics* 33: 2938-2940. https://doi.org/10.1093/bioinformatics/btx364

28. Bastian, Mathieu, Sebastien Heymann, Mathieu Jacomy. 2009. 'Gephi: an open source software for exploring and manipulating Networks', *Third International ICWSM Conference*.

29. Gu, Ziguang, Roland Eils, Matthias Schlesner. 2016. “Complex heatmaps reveal patterns and correlations in multidimensional genomic data.” *Bioinformatics* 32: 2847-2849. https://doi.org/10.1093/bioinformatics/btw313

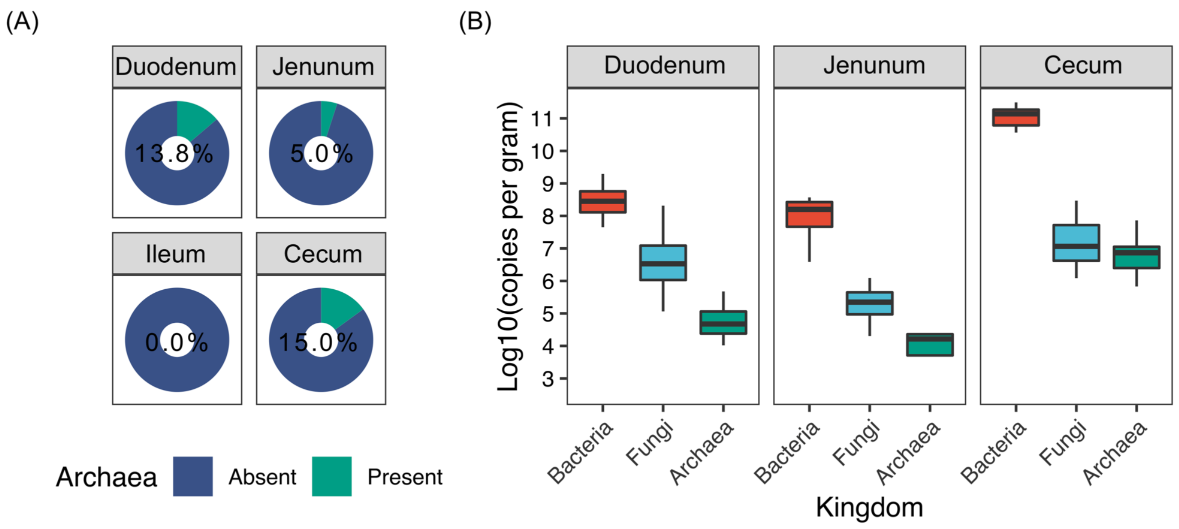

Figure S1 The archaeal community in the chicken gut. (A) Prevalence of archaea in the gut four segments. (B) Absolute abundance of archaea in the gut compared with bacteria and fungi.

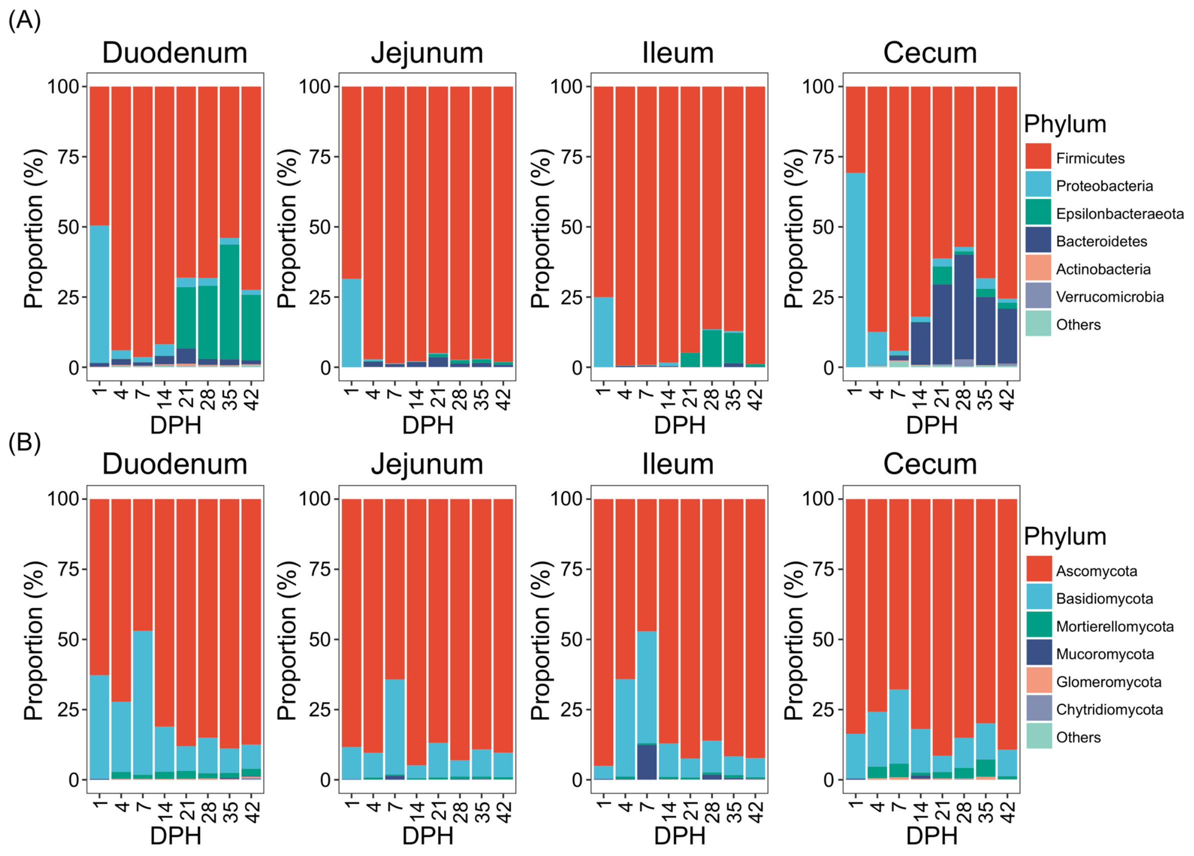

Figure S2 Microbial community composition of the chicken gut microbiota at the phylum level.

Composition of bacterial (A) and fungal (B) communities.

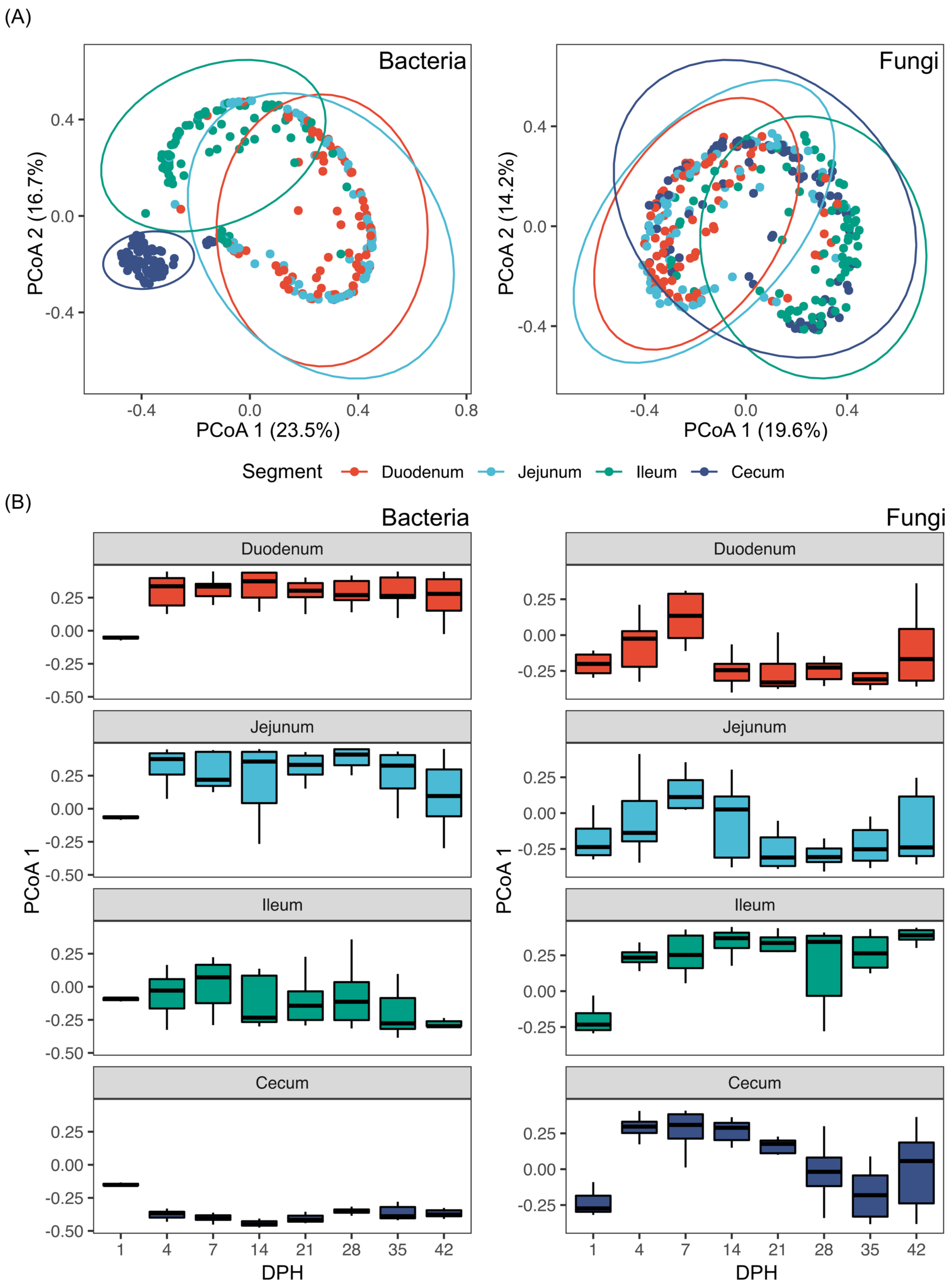

Figure S3 Beta-diversity of the chicken gut microbiota based on quantitative microbiome profiling (QMP). (A) PCoA of Bray-Curtis dissimilarity of absolute abundances of bacterial and fungal

taxa. Different colors represent samples from different segments. (B) Values of the first PCoA axis in different segments of bacterial and fungal communities over time.

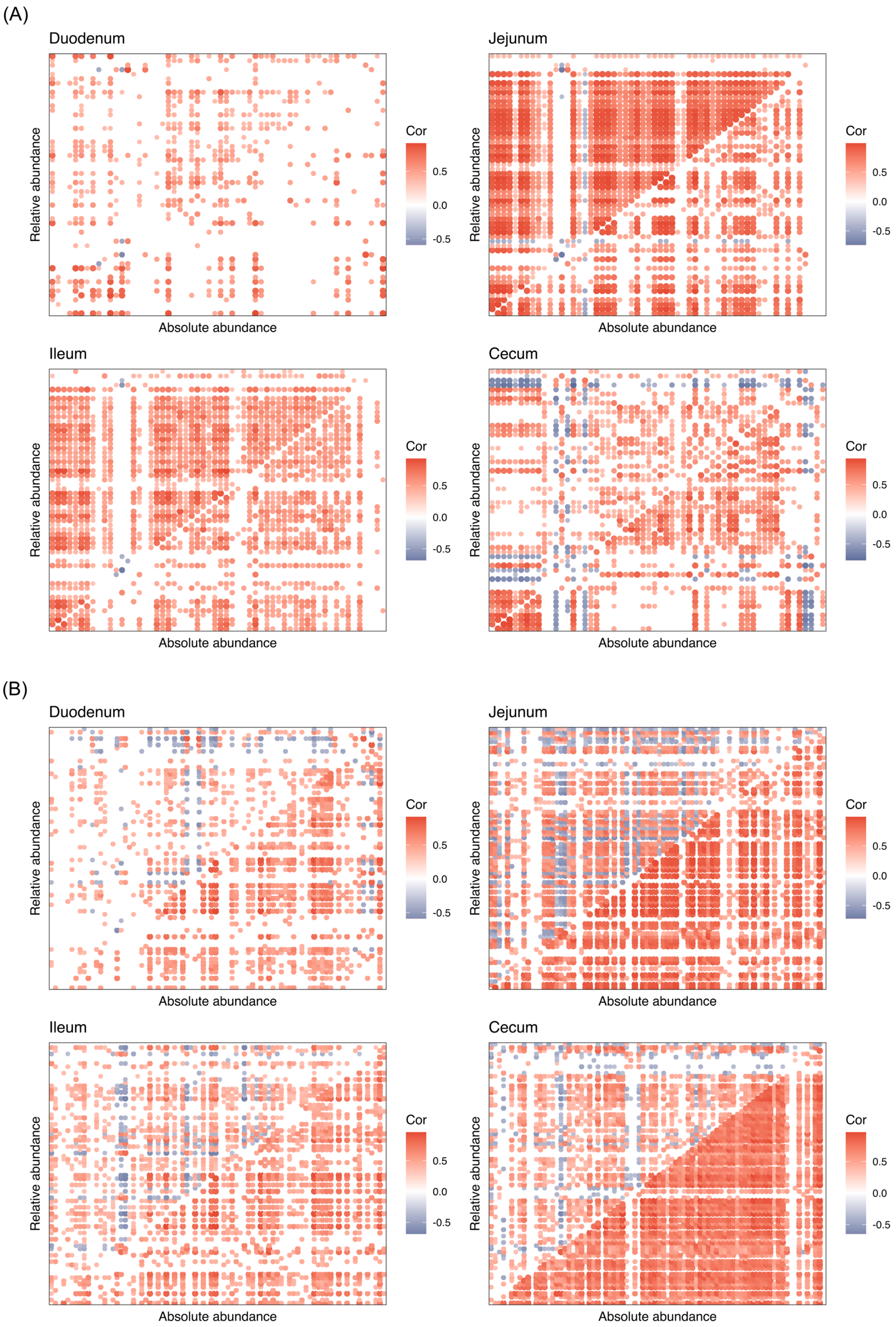

Figure S4 Pairwise correlations among microbial taxa abundance in QMP and relative microbiome profiling (RMP). (A) Pairwise correlations among bacterial taxa abundance in QMP and

RMP. (B) Pairwise correlations among fungal taxa abundance in QMP and RMP. Red nodes: positive correlations (adjusted *P* values < 0.05); blue nodes: negative correlations (adjusted *P* values < 0.05); white nodes: nonsignificant (adjusted *P* values > 0.05).

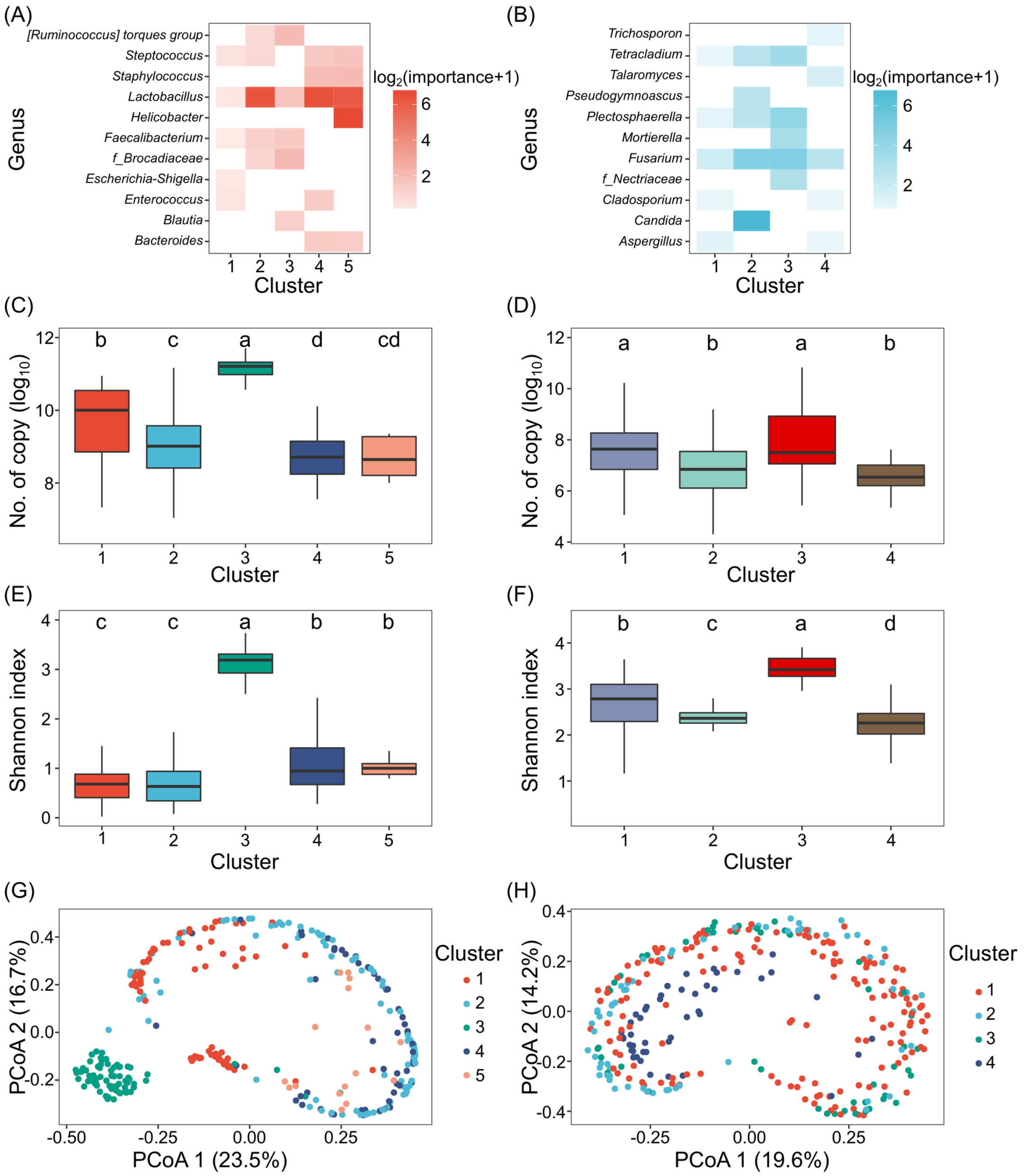

Figure S5 Microbial load and diversity of different DMM clusters. Taxa that contributed the most to the accuracy of the DMM for each cluster of bacteria (A) and fungi (B). The importance values were log-transformed. The bacterial (C) and fungal (D) loads in each cluster. The microbial load was measured by the copy number of the marker genes (16S and ITS). Shannon index of the bacterial communities (E) and fungal communities (F) in each cluster. PCoA of Bray-Curtis dissimilarity of absolute abundances of bacterial (G) and fungal (H) taxa.

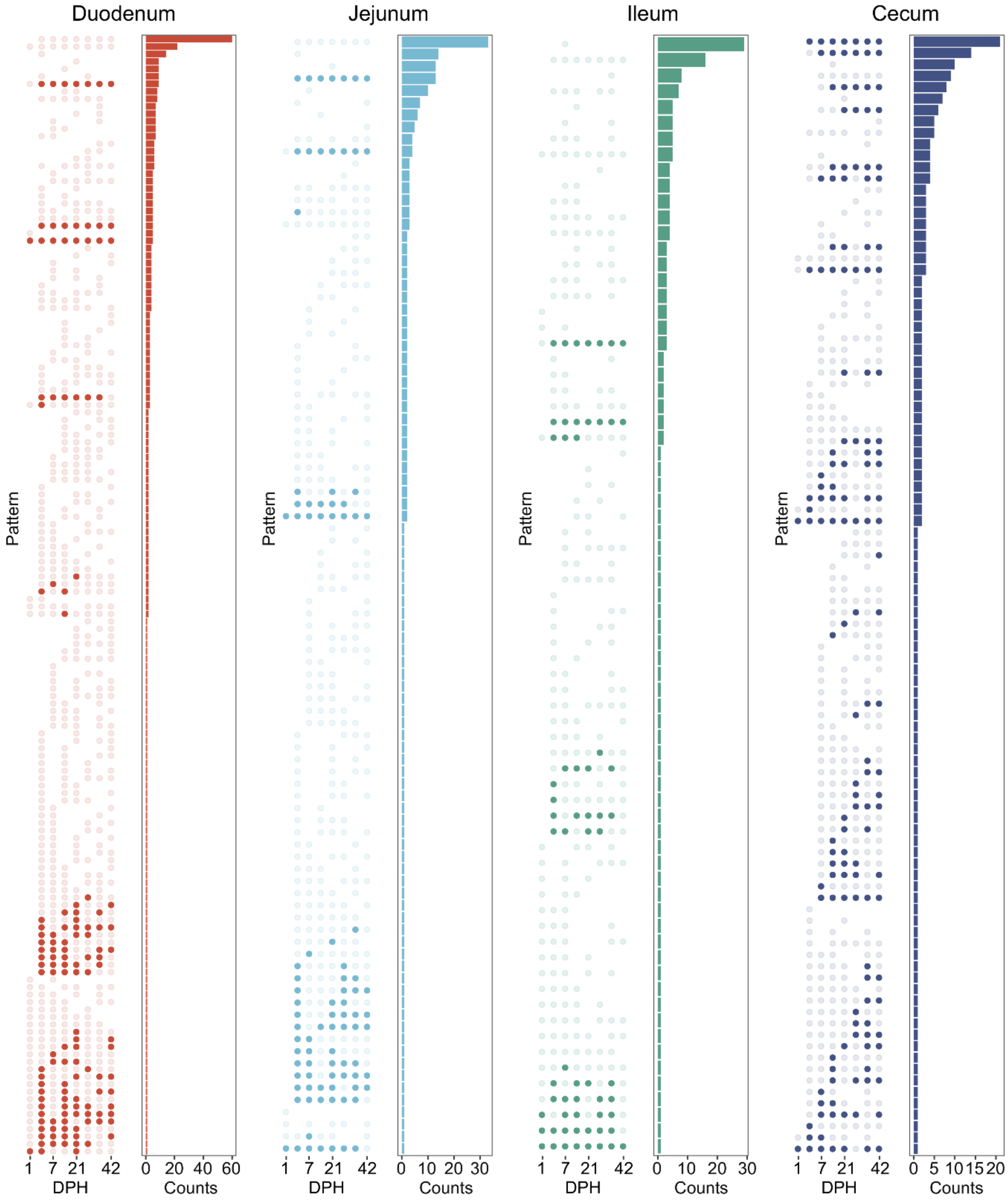

Figure S6 Longitudinal occurrence patterns of the bacterial genera in the chicken gut. A total of 529 genera at eight time points were used to summarize the occurrence patterns in the four gut segments. Dark points represent the presence of taxa (n ≥ 9); white points represent the absence of taxa; light points represent the transition between "presence" and "absence". The length of the bar represents the counts of each pattern.

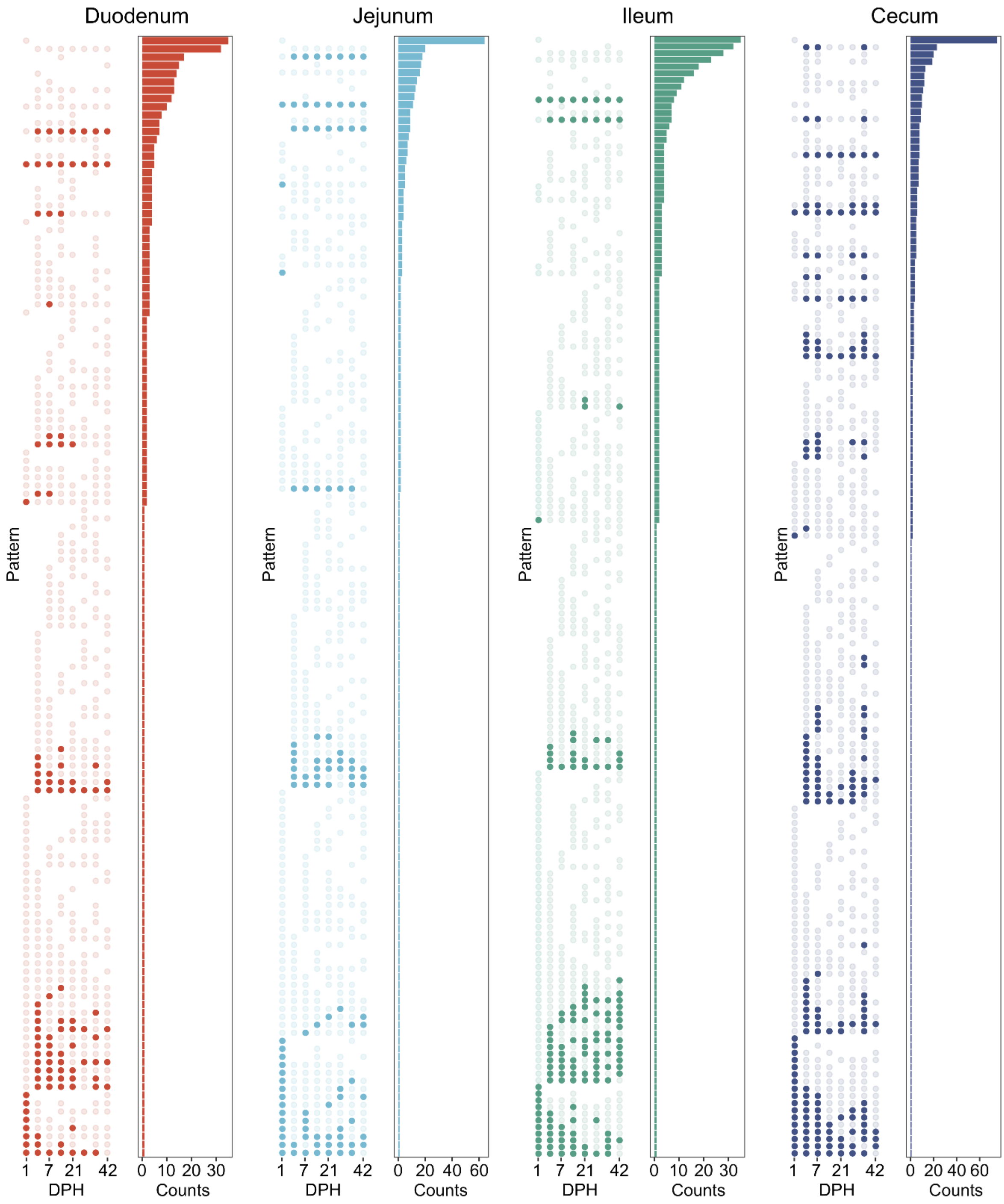

Figure S7 Longitudinal occurrence patterns of the fungal genera in the chicken gut. A total of 603 genera at eight time points were used to summarize the occurrence patterns in the four gut segments. Dark points represent the presence of taxa (n ≥ 9); white points represent the absence of taxa; light points represent the transition between "presence" and "absence". The length of the bar represents the counts of each pattern.

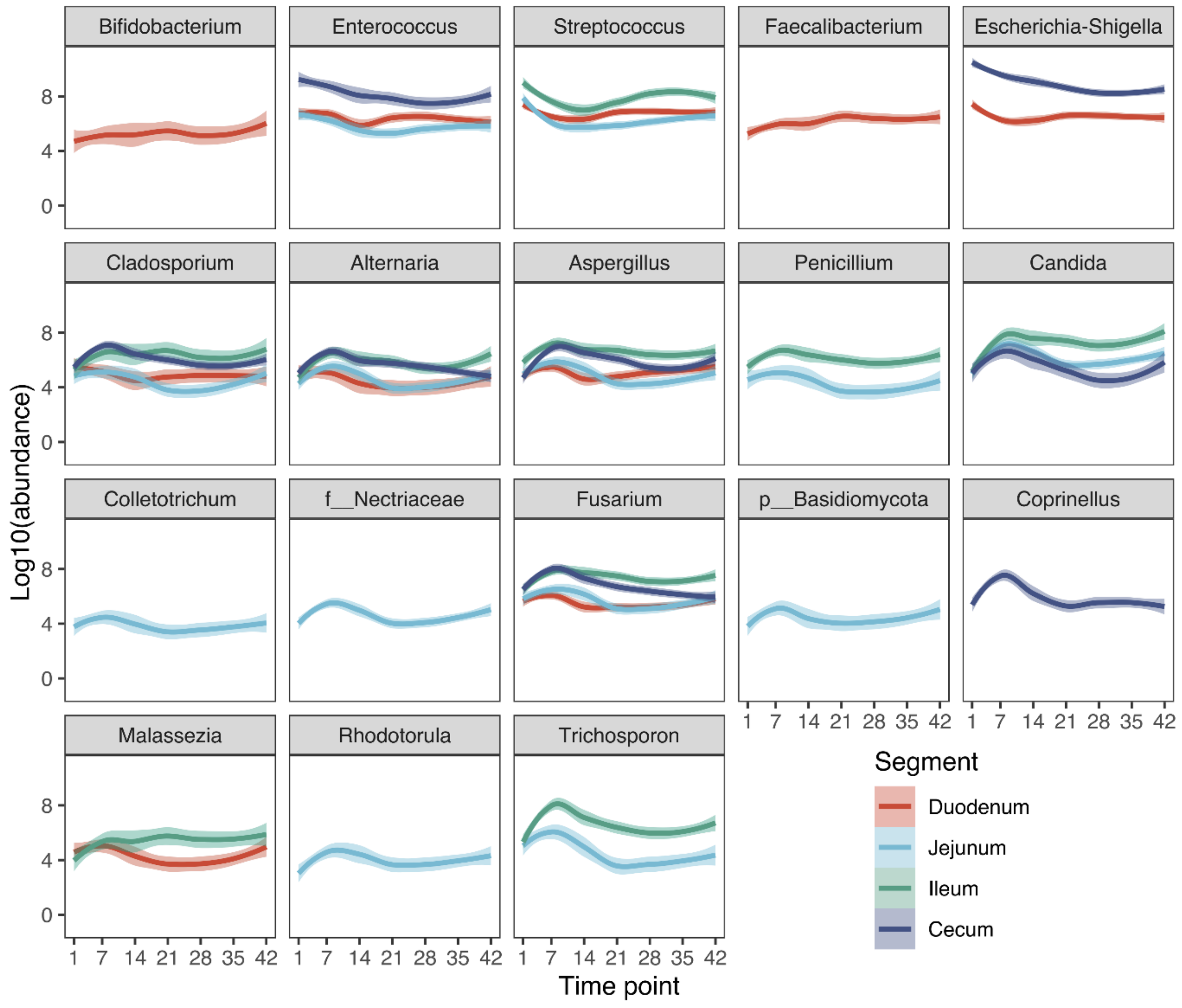

Figure S8 Absolute abundance of the genera belonging to the "core" pattern. The changes in the absolute abundance of the 18 genera through the 42 days. The missing line indicates that this taxon is not the "core" genus in the corresponding segment.

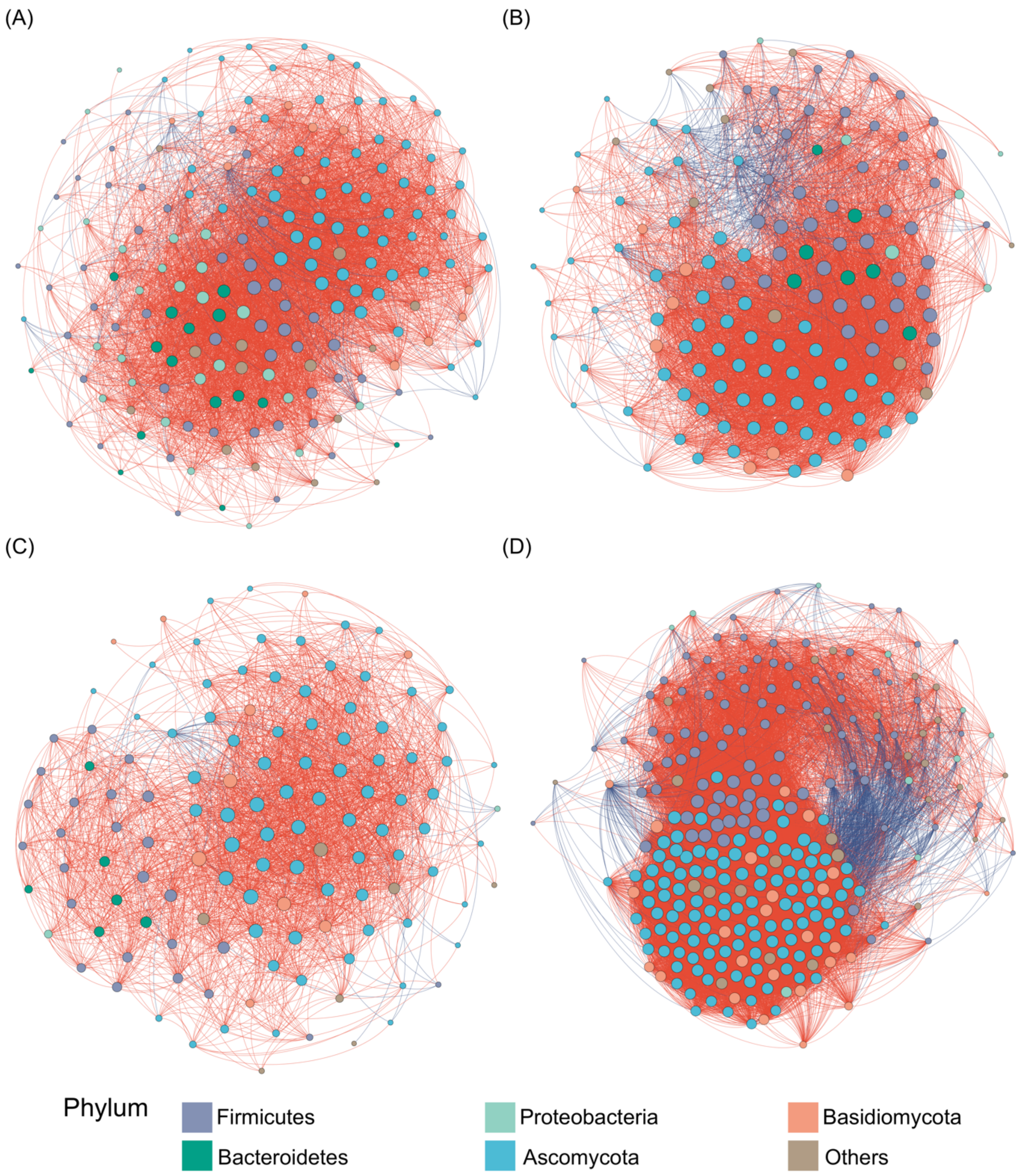

Figure S9 The four submicrobial networks generated from the genera in different gut segments. Microbial cooccurrence network in the duodenum (A), jejunum (B), ileum (C), and cecum (D). Red lines represent positive correlations; blue lines represent negative correlations; nodes with the same color belong to the same phylum.

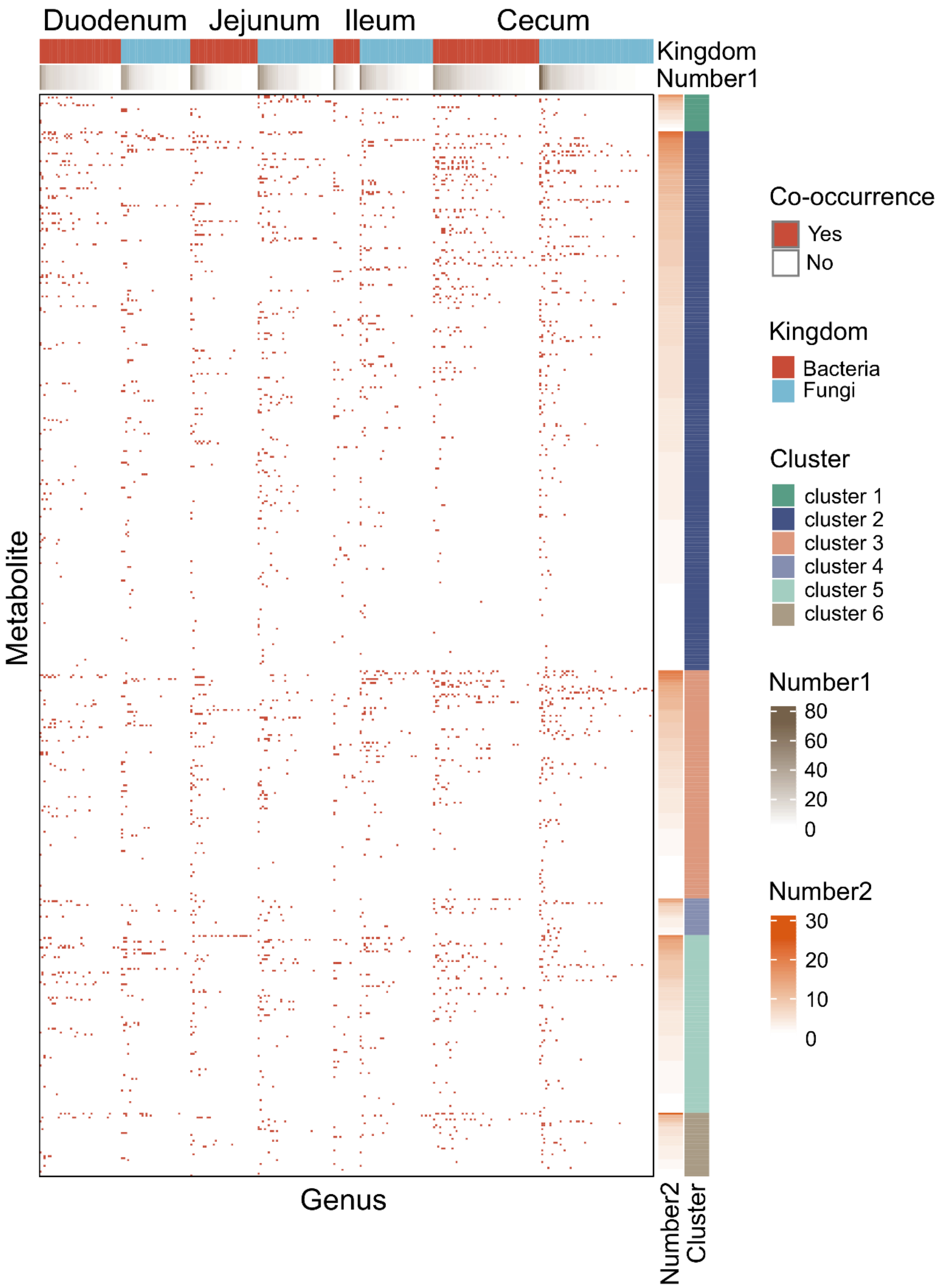

Figure S10 Cooccurrence profiles of microbes and metabolites. Heatmap of the cooccurrence profiles of microbes from different segments and metabolites from different clusters. Red cells represent the top 1% cooccurrence possibilities; number 1: the number of metabolites associated with the genera; number 2: the number of genera associated with the metabolites.

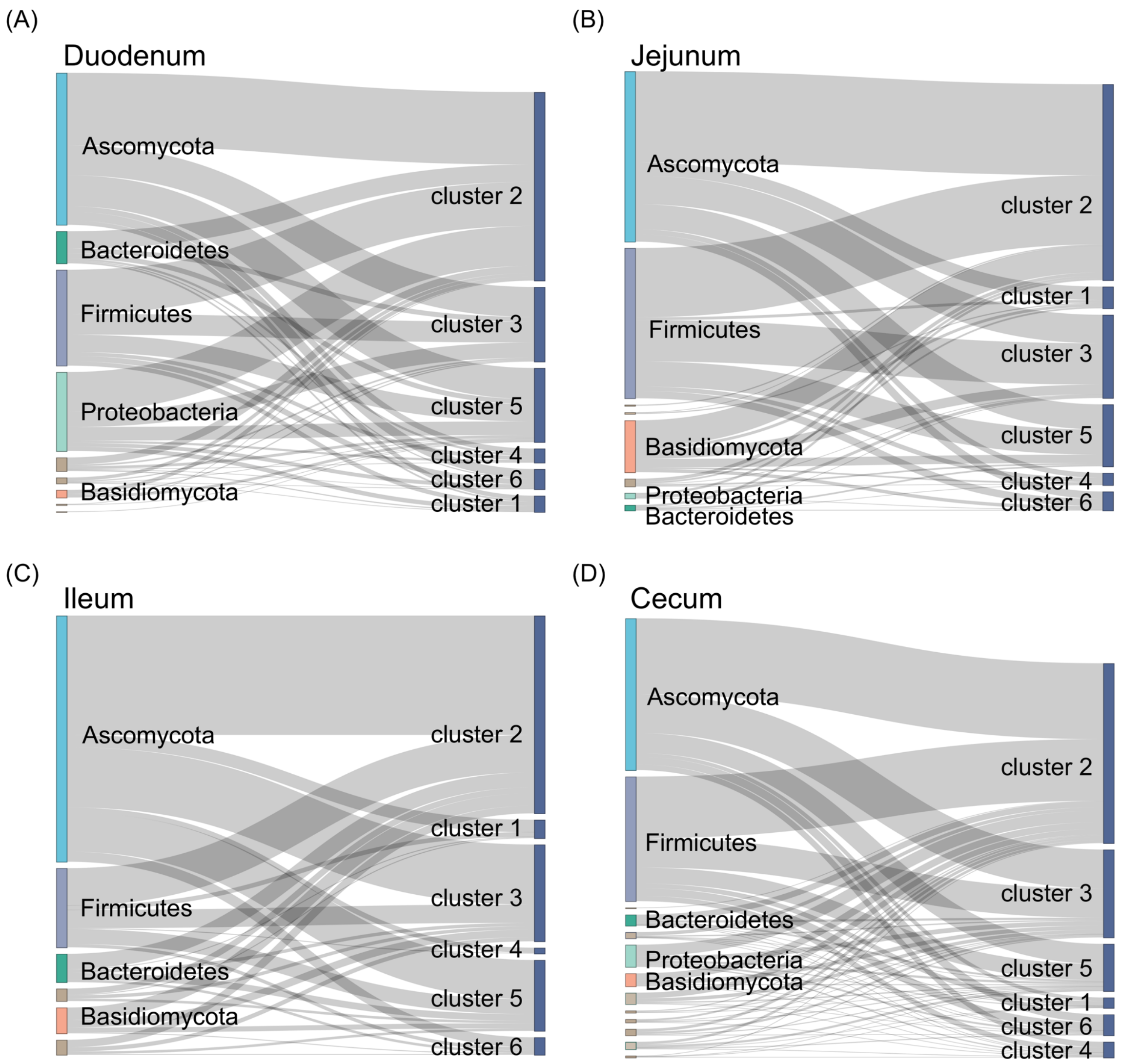

Figure S11 Associations between genera in different phyla and metabolites in different clusters discovered using mmvec. (A) duodenum, (B) jejunum, (C) ileum, and (D) cecum. The height of the bar represents the number of genera or metabolites.
